# Novel 3D Flipwell system that models gut mucosal microenvironment for studying interactions between gut microbiota, epithelia and immunity

**DOI:** 10.1038/s41598-023-28233-8

**Published:** 2023-01-17

**Authors:** Maria A. Beamer, Cassandra Zamora, Andrea L. Nestor-Kalinoski, Veani Fernando, Vandana Sharma, Saori Furuta

**Affiliations:** 1 Department of Cell & Cancer Biology, College of Medicine and Life Sciences, University of Toledo Health Science Campus, 3000 Arlington Ave., Toledo, OH 43614 USA; 2 Division of Pediatric Rheumatology, Department of Pediatrics, University of Michigan, Ann Arbor, MI 48109 USA; 3 College of Natural Sciences and Mathematics Instrumentation Center, University of Toledo, 3100 West Towerview Blvd, Bowman-Oddy 1073, Toledo, OH 43606 USA; 4 Department of Surgery, College of Medicine and Life Sciences, University of Toledo Health Science Campus, 3000 Arlington Ave., Toledo, OH 43614 USA; 5 MetroHealth Medical Center, Case Western Reserve University School of Medicine, Case Comprehensive Cancer Center, 2500 MetroHealth Drive, Cleveland, OH 44109 USA

**Keywords:** Biological techniques, Biotechnology, Cancer, Cell biology, Drug discovery, Immunology, Microbiology

## Abstract

Gut mucosa consists of stratified layers of microbes, semi-permeable mucus, epithelium and stroma abundant in immune cells. Although tightly regulated, interactions between gut commensals and immune cells play indispensable roles in homeostasis and cancer pathogenesis in the body. Thus, there is a critical need to develop a robust model for the gut mucosal microenvironment. Here, we report our novel co-culture utilizing 3D Flipwell system for establishing the stratified layers of discrete mucosal components. This method allows for analyzing synchronous effects of test stimuli on gut bacteria, mucus, epithelium and immune cells, as well as their crosstalks. In the present report, we tested the immuno-stimulatory effects of sepiapterin (SEP, the precursor of the cofactor of nitric oxide synthase (NOS)—BH_4_) on the gut mucosal community. We previously reported that SEP effectively reprogrammed tumor-associated macrophages and inhibited breast tumor cell growth. In our co-cultures, SEP largely promoted mucus integrity, bacterial binding, and M1-like polarization of macrophages. Conversely, these phenomena were absent in control-treated cultures. Our results demonstrate that this novel co-culture may serve as a robust in vitro system to recapitulate the effects of pharmacological agents on the gut mucosal microenvironment, and could potentially be expanded to test the effects outside the gut.

## Introduction

Each human body harbors about 40 trillion microbes—collectively termed the microbiome—reaching the number equivalent to that of host cells (30 trillion)^1^. These commensals are the most profuse in the gut, weighing up to 1 kg per person^2^. They are not merely passive passengers, but essential for body’s fundamental functions, including tissue/organ development, immunity, and metabolism^3,4^. Guts provide the largest contact surface for microbes and immune cells, where immune cells execute defense against pathogens, but also establish tolerance towards harmless symbionts^5,6^. A single layer of epithelium separates the lumen from underlying tissues and restricts permeability through tight junctions. The epithelium is then shielded by dense, selectively permeable mucus—a glycoprotein network abundant in mucin-2 (MUC2)—that compartmentalizes microbes and immune responses^5^. MUC2 is produced by goblet cells in the crypts of the epithelium and upregulated by the presence of microbiota, serving as a protective shield^7,8^. The mucus also harbors different antibacterial molecules, such as defensins, lysozyme and phospholipase A2-IIA, which kill penetrating bacteria^7^. Furthermore, invading bacteria are engulfed by dendritic cells (DCs), which in turn promotes IgA production by B cells (plasma cells) allowing for opsonization of the target cells^9^. Conversely, subsets of DCs could be conditioned by MUC2 to become tolerogenic, transporting live commensals to lymphoid tissues to establish tolerance^10–13^.

Gut microbe-immune cell interactions play critical roles in prevention/treatment of various diseases, in particular cancer. This feat is mostly ascribed to their reciprocal communications, including anti-microbial peptides and bacterial products, namely, PAMPs (pathogen-associated molecular patterns) and MAMPs (microbe-associated molecular patterns), which promote immune cell activities^7,14^. Nevertheless, microbe-immune cell interactions are largely impacted by host metabolism, diet, diseases, and drug treatment, potentially leading to dysbiosis—an imbalance in the composition of the microflora^15–17^. Certain patients could be benefited by transplanting fecal microbiota from healthy donors^18^. Screening for specific stimuli impacting microbe-immune cell interactions would be greatly assisted by a robust model system.

Here, we present our novel co-culture system intended to recapitulate the gut mucosal microenvironment. Our co-culture consists of stratified layers of commensal bacteria, mucus, intestinal epithelium and immune cells. We tested the pro-immunogenic effect of sepiapterin (SEP), the endogenous precursor of BH_4_ (tetrahydrobiopterin)—cofactor of nitric oxide synthase (NOS), which we previously showed exerts pro-immunogenic activities on breast tumor microenvironment^19,20^. SEP could effectively reprogram tumor associated macrophages from the immuno-suppressive M2-type to immuno-stimulatory M1-type, while also strongly suppressing the growth of breast tumor cells^19,20^. We show here that SEP, but not control treatment, dramatically elevated mucus production by colon epithelium, which caused bacterial entrapment within the mucus and increased MUC2 expression to augment the mucosal integrity. SEP also induced M1-like polarization of naïve macrophages in co-cultures, unlike control treatment. These observations support the validity of the new co-culture system to mimic the gut mucosal microenvironment.

## Results

### Novel multi-cellular 3D co-culture system to model gut mucosal microenvironment

**Figure 1:**
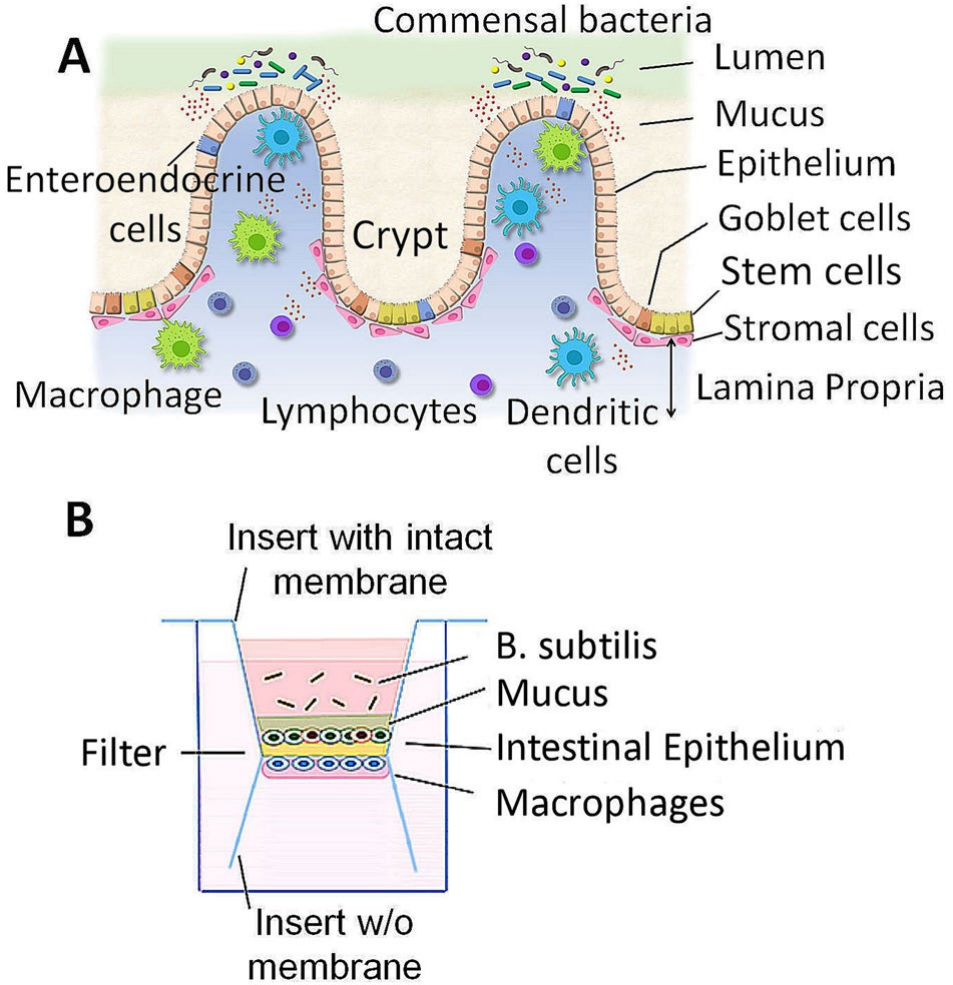
Gut mucosa and the respective co-culture system. (**A**) Simplified scheme of gut mucosal microenvironment. (**B**) Scheme of the co-culture system to recapitulate the gut mucosa.

**Figure 2:**
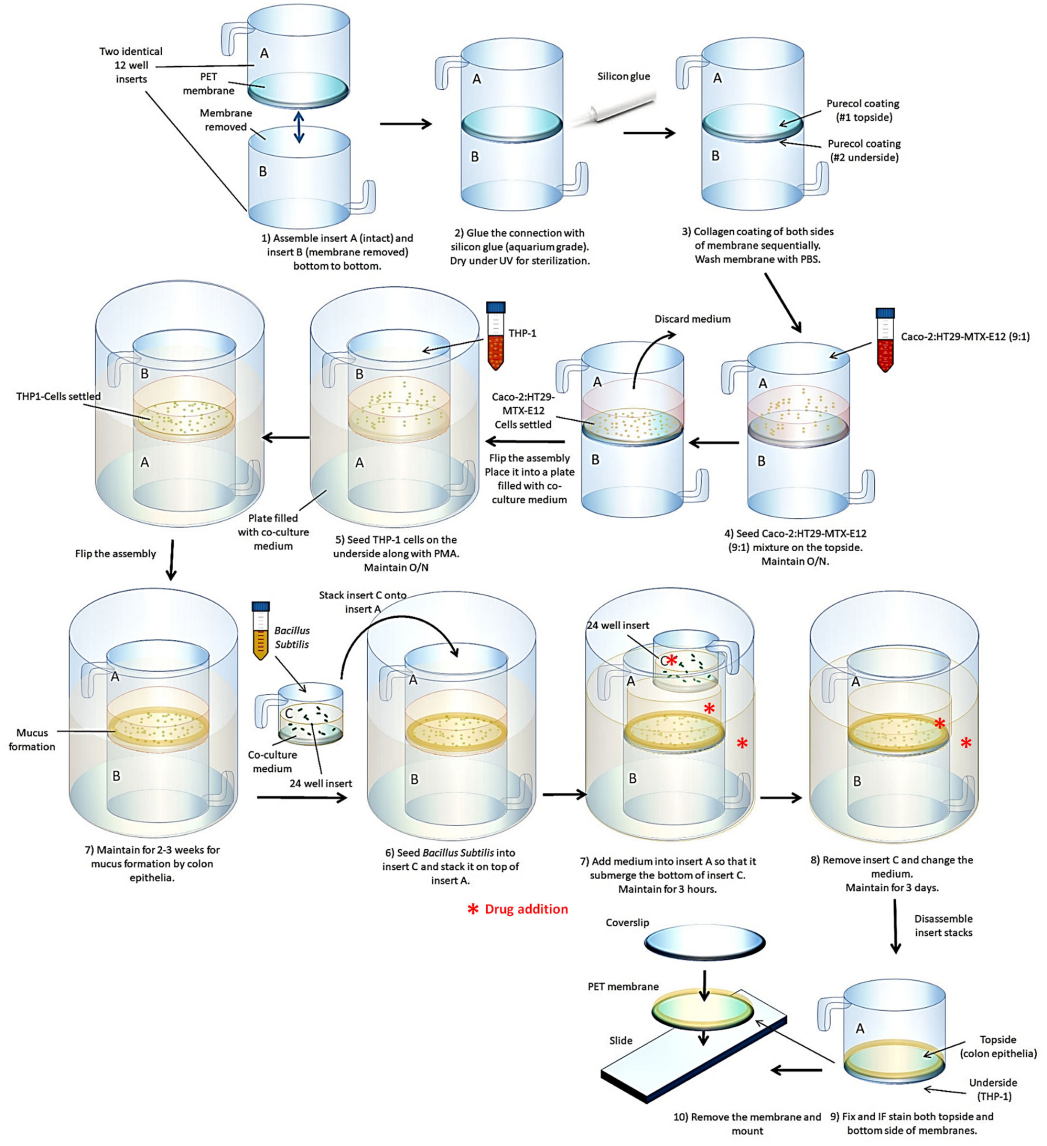
Schematic of the 3D Flipwell assembly and co-culture method: 3D Flipwell construction, co-culturing and analyses. Note that these steps exclude experiment specific steps of permeabilization, fixation, and immunofluorescent staining or dehydration steps for electron microscopy. Red asterisks: points of drug addition.

**Figure 3:**
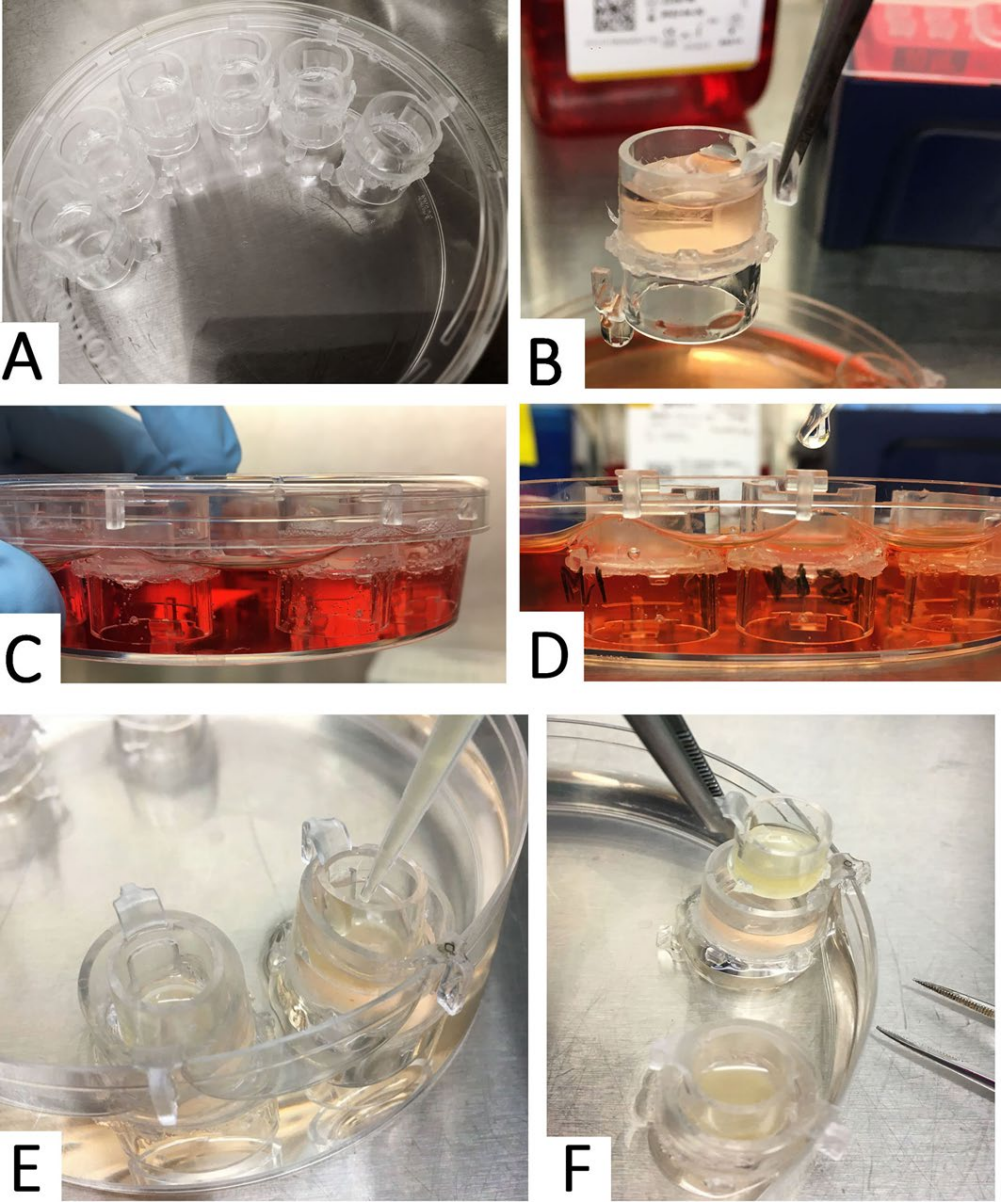
3D Flipwell construction and culturing. (**A**) Constructed 3D Flipwells after two inserts were glued together with a silicone sealant and membranes are coated with PureCol. Flipwells were spaced along the rim of a deep-well petri-dish. (**B**) Flipwells were seeded with cells on one side. (**C**) Top inserts (topside of the membrane) were seeded with Caco2/HT-29 cells and maintained until cells settled. (**D**) Flipwells were flipped and the bottom inserts (underside of the membrane) were seeded with non-adherent THP-1 monocytes. Cells were treated with PMA for differentiation to adherent macrophages. (**E**) After cell attachment, Flipwells were flipped to have colon epithelial cells faced upwards so that they produced overhanging mucus in 2–3 weeks. A smaller insert containing bacteria was added onto the insert with colon epithelial cells and left for 3 h. (**F**) Bacterial insert was removed and co-culture was left for 3 days with daily medium change.

Gut mucosa is composed of stratified layers of gut microbes in the lumen, mucus, intestinal epithelium with apical microvilli, and stroma that contains a large number of immune cells (Fig. 1A). We have developed a novel multicellular 3D co-culture (we termed the 3D Flipwell) system to model the gut mucosal microenvironment (Fig. 1B) by modifying the system initially developed by Noel et al.^21^. Our new method utilizes bottom-to-bottom stacked cell culture inserts sandwiching a single layer of PET membrane (0.4 μm pore size). A mixture (9:1) of colon epithelial Caco2 and mucus-producing HT-29/MTX cells were seeded on one side of the membrane, whereas THP-1 monocytes were seeded on the other side of the membrane. These cells were co-cultured to synchronously undergo colon epithelial cell polarization, mucus formation, macrophage differentiation and polarization (Figs. 2, 3). After the establishment of the stratified layers, another cell culture insert that included commensal bacteria, *Bacillus subtilis*, was added onto the co-culture for 3 h and removed (Figs. 2, 3). Cultures were allowed to recover for 3 days. The membranes were removed and processed for confocal and SEM imaging (Fig. 2).

### SEP treatment of co-cultures elevated the thickness and integrity of mucus and promoted M1-type polarization of macrophages

Experimental drugs could be added to the growth medium of co-cultures to monitor the synchronous effects on bacteria, mucus, gut epithelial and immune cells, as well as crosstalks between them. Here, we tested the effects of SEP, an endogenous precursor of BH_4_—the cofactor of NOS^22^. We previously showed that SEP could improve the immunogenicity of breast tumor microenvironment^19,20^. SEP effectively reprograms tumor associated macrophages from the immuno-suppressive M2-type (alternatively activated) to immuno-stimulatory M1-type (classically activated), while also strongly suppressing breast tumor cell growth^19,20^. Here, we tested the effects of SEP on colon epithelial Caco2 and mucus-producing HT-29/MTX cells, THP-1 macrophages and Gram-positive commensal *Bacillus subtilis*, where SEP could activate both mammalian and bacterial NOS isoforms to produce NO^23^. We also made comparisons between mono-cultures and co-cultures.

**Figure 4:**
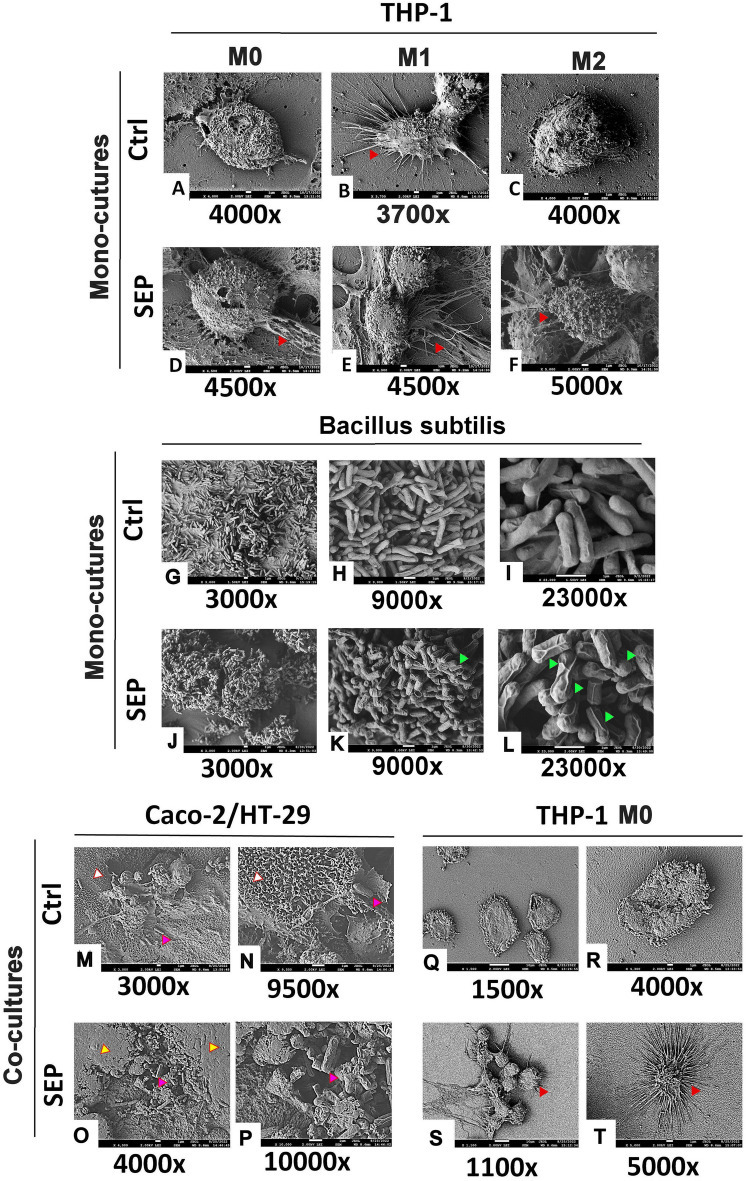
Scanning electron microscopy (SEM) images of Caco2-HT29 epithelial layer and THP1 cells cocultured on the opposite side of the 3D Flipwell membrane. (**A–C**) THP-1 cells in different polarization stages: M0 (naïve, **A**), M1 (classically activated, **B**) and M2 (alternatively activated, **C**). Note the elongated pseudopods of M1 cells, while they are absent in M0 and M2 cells. (**D–F**) M0, M1 and M2 macrophages treated with SEP (100 µM). Note the emergence of M1-like elongated structures in M0 and M2 cells. Solid red arrows indicate elongated pseudopods typical of the M1-like phenotype. (**G–I**) Control-treated *B. subtilis*. (**J–L**) SEP-treated *B. subtilis*. Solid green arrows point to wrinkled morphology of SEP-treated bacteria. (**M,N**) Control-treated Caco-2/HT29 epithelial cells in co-cultures at different magnifications. Solid pink arrows indicate *B. subtilis*, while solid white arrows point to microvilli. (**O,P**) SEP-treated Caco-2/HT29 cells in co-cultures at different magnifications. Solid pink arrows indicate *B. subtilis*, while solid yellow arrows point to vast and thick mucosal secretions that are absent in the untreated control. Microvilli are submerged in mucus and not easily seen. Here Bacteria are present in aggregates within thick mucus unlike the untreated control. (**Q,R**) Control-treated M0-type macrophages in co-cultures at different magnifications. (**S,T**) SEP-treated M0-type macrophages in co-cultures at different magnifications. Note the emergence of M1-like elongated structures in co-cultured M0 cells. Solid red arrows indicate elongated pseudopods typical of the M1-like phenotype. NOTE that *B. subtilis* was cultured in LB and added to the epithelial side of the Flipwell in the smaller transwell insert for several hours of co-culture. It was later added to the epithelial cells for imaging and visualization of bacterial clustering in mucus. The original raw images are included in the Supplementary Information file.

Mono-cultured THP-1 macrophages captured by scanning electron microscopy (SEM) exhibited a clear morphological difference of M1-type compared to M0 (naïve) and M2 types. Consistent with a previous report^24^, M1-type macrophages showed elongated, spindle-like structures with prominent pseudopods, unlike spherical, smoother structures of M0 and M2 types (Fig. 4A–C). After SEP treatment, however, both M0 and M2 types exhibited spindle-like structures comparable to M1 type (Fig. 4D–F). Such morphological shift of M0 and M2 macrophages after SEP treatment well supported our earlier finding that SEP caused their functional reprogramming to M1 type^19^. SEM imaging of control-treated *Bacillus subtilis* in mono-cultures (before addition to co-cultures) showed smooth, rod-shaped structures with single polar flagella (Fig. 4G–I). However, SEP-treated bacteria exhibited wrinkled, rougher surface, indicating a morphological change (Fig. 4J–L). In fact, such wrinkled morphology is likely to be the result of biofilm formation as reported^25^, which partly accounts for the roles of these bacteria in disease prevention and better health of the host organisms^26,27^. Consistently, increased NO levels are found to induce biofilm formation by *Bacillus subtilis*^23,28^.

In our co-cultures, control-treated colon epithelia (consisting of Caco2 and HT-29/MTX cells) showed prominent microvilli on the surface (Fig. 4M,N). In contrast, SEP-treated colon epithelia showed a dense, agglutinative mucus on top of the epithelium. Some areas of mucus showed bacterial clustering and entrapment (Fig. 4O,P). These observations are consistent with previous reports that increased NO levels and gut bacteria could elevate mucus production by colon epithelia as part of mucosal defense mechanism^7,29^. Our co-cultures also included naïve (M0) THP-1 macrophages. In control-treated samples, these macrophages maintained spherical, smooth morphology identical to mono-cultured M0-type cells (Fig. 4A,Q,R). In contrast, after SEP treatment, these macrophages acquired spindle-like structures with numerous pseudopods, similar to M1-type macrophages (Fig. 4B,S,T)^24^. This observation was consistent with previous reports that high doses of certain probiotics, such as *Bacillus subtilis*, could induce M1 polarization of macrophages^30,31^. These results suggest that our co-culture system could reproduce the synchronous responses of gut bacteria, mucus, colon epithelia and immune cells to an exogenous stimulus as well as crosstalks among them.

### SEP treatment elevated mucosal protein (MUC2) production by the gut epithelium and M1-type markers of macrophages in co-cultures

**Figure 5:**
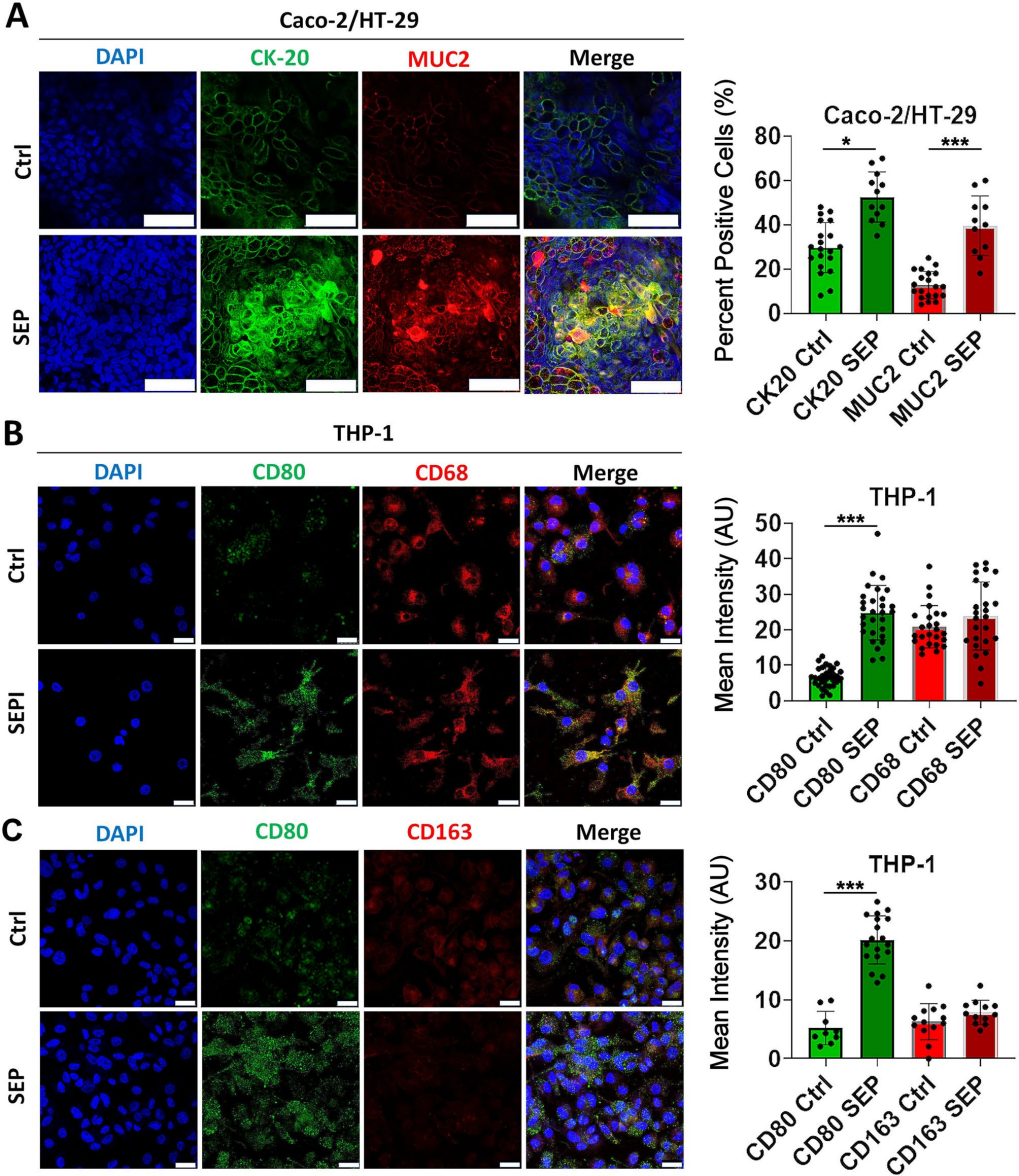
Sepiapterin promotes MUC2 and Cytokeratin-20 expression in Caco-2:HT-29 epithelial layer and the emergence of M1-like phenotype in naïve THP-1 macrophages in 3D Flipwell co-cultures. (**A**) (Left) Confocal images of Caco-2:HT-29 cells co-cultured with THP-1 cells in the 3D Flipwells and stained for CK20 (green) and MUC2 (red). Cytokeratin staining is very pronounced in the areas surrounding mucus production as indicated by MUC2 expression. Scale bar represents 50 µm. The original raw images are included in Supplementary Information file. (Right) Percent positive cells for CK20 and MUC2. (**B**) (Left) Confocal images of THP-1 cells cocultured with Caco-2:HT-29 in the 3D Flipwells and stained for Pan Macrophage marker CD68 (red) and CD80 (green). Pan Macrophage marker CD68 is present in both SEP treated and untreated control samples. CD80, typical of the M1 macrophage phenotype, is more pronounced in SEP treated sample with long pseudopods extending outward typical of the M1 phenotype. (Right) Mean intensities of CD68 and CD80. (**C**) (Left) Confocal images of THP-1 cells cocultured with Caco-2:HT-29 in the 3D Flipwell and stained for Pan Macrophage marker CD80 (green) and CD163 (red) to validate phenotypic polarization toward M1 phenotype and away from M2 phenotype. Scale bar represents 25 µm. The original raw images are included in Supplementary Information file. (Right) Mean intensity of CD80 and CD163. Error bars: mean ± STDEV. *p < 0.05; ***p < 0.001.

To quantify phenotypic changes of co-cultured cells in response to an exogenous stimulus, expression of specific markers could be analyzed by confocal imaging. Our confocal microscopy showed that SEP treatment caused a substantial increase in the expression of a mucus protein, MUC2, and epithelial marker, CK20, in mucus/colon epithelial layers. The expression patterns of both proteins were partially overlapped or in close proximity, suggesting their positive correlation (Fig. 5). Furthermore, after SEP treatment, M0 THP-1 macrophages exhibited a dramatic increase of a M1 marker CD80, but no changes in a M2 marker CD163 and a pan-macrophage marker CD68, attesting to their M1-like polarization (Fig. 5). These findings further point to the utility of our new co-culture system in studying individual signaling events in different components of the gut mucosal microenvironment in response to an external stimulus.

## Discussion

Gut microbiota and immune system reciprocally interact to maintain the whole body’s homeostasis. In the gut mucosa, direct interactions of gut microbes and immune cells are mostly precluded by the barrier formed by stratified layers of mucus and intestinal epithelium^32^. Nevertheless, gut mucosal DCs occasionally move across the barrier to sample commensals either for local immune responses or for transporting them to distant tissues^9,12,33^. Such mucosal environment and microbe-immune cell interactions are conserved throughout metazoans, attesting to their indispensable roles in the survival of all animals^34^. Any external stimuli that augment or disrupt the mucosal homeostasis would have substantial impacts on the health of organisms^32^. To determine the impact of a specific agent on gut mucosal community and disease pathogenesis, it is essential to develop a robust model system that recapitulates the gut mucosal microenvironment.

Gut-on-chip system developed by Donald Ingber’s group^35^ has gained traction in the past decade. This system consists of a microfluidic device that allows reconstruction of the mechanical and physiological properties of the gut epithelium and the microenvironment^35^. Such gut-on-chip system could be utilized to establish the 3D architecture of colon epithelium and to co-culture colon epithelium with bacteria and immune cells, as well as other types of cells^35,36^. Nevertheless, this system succumbs to certain inherent limitations. First, fabrication of this system remains complex and labor-intensive, limiting the throughput of the technology^36^. Second, chip materials (namely, polydimethylsiloxane (PDMS)) adsorb a range of molecules, altering their actual concentrations in the media and impairing pharmaceutical analyses^36^. Noel et al. recently reported another co-culture system that is relatively easy to fabricate and effectively models the gut mucosal microenvironment. In their system using a single culture insert, colon epithelium was seeded on one side of the membrane, whereas pre-differentiated adherent macrophages were seeded on the other side of the membrane^21^. Despite the great advantage of this new co-culture system, their system requires pre-differentiation and chemical dissociation of macrophages from another vessel, which could potentially damage the integrity of cells. In the present report, we thus engineered a stacked dual-insert system that enables seeding colon epithelial cells on one side and undifferentiated non-adherent monocytes on the other side of the membrane (Fig. 3). This new co-culture system allows for the synchronous progresses of colon epithelial polarization, mucus formation, and monocyte-macrophage differentiation and polarization within a single co-culture, eliminating the need to detach and re-plate pre-differentiated macrophages. Besides, fabrication of our system is easy and does not require special materials that could impact analyses. Thus, this co-culture model could be utilized for high throughput analyses.

Our results demonstrate that our co-culture system was able to establish the stratified layers of MUC2-rich mucus, intestinal epithelium with prominent microvilli, and well-differentiated macrophages. Additionally, we tested the pro-immunogenic effect of SEP, an endogenous precursor of BH_4_—the cofactor of multiple enzymes including NOS and aromatic amino acid hydroxylase^22^. SEP was originally discovered as a yellow eye pigment of Drosophila melanogaster^37^. SEP is ubiquitously synthesized in the body from bacteria to mammals, as part of the salvage pathway of BH_4_ synthesis^38^. In clinical studies, SEP supplement has been tested for the treatment of BH_4_ deficiency-linked chronic disorders such as phenylketonuria and cardiovascular diseases^39,40^. Supplementing SEP is found to be more effective than supplementing BH_4_ itself in elevating BH_4_ levels in the body^41^. We previously reported that SEP could induce M2-to-M1 reprogramming of tumor-associated macrophages, leading to a dramatic suppression of breast tumor cell growth^19,20^. We show here that SEP induced naïve M0 macrophages to acquire M1-like phenotype in co-cultures, in contrast to control treatment which failed to do so. Furthermore, SEP markedly elevated the thickness of mucus and promoted the mucus/epithelial integrity, as indicated by the significant increase in MUC2 and CK20 levels. This observation is in line with a previous report that NO stimulates mucus production by the epithelium of the gastrointestinal tract as a mucosal defense mechanism^29^. We indeed saw that the enhanced mucus production by SEP treatment helped promote binding of bacteria to the mucosal surface. This would in turn induce MUC2 expression by epithelium, further promoting the barrier function^11,42^. In addition, SEP treatment induced morphological change of gut commensal, *Bacillus subtilis*, which was implicated in elevated host defense mechanism^26,27^. These observations strongly support the validity of our new co-culture system to model the gut mucosal microenvironment and synchronous responses of different components to an external stimulus.

While the present study specifically focuses on the utility of our new co-culture system to model gut mucosal community, this system could also be utilized to investigate how mucosal immunity influences the rest of the body. Gut mucosal immune cells, in particular DCs, are stimulated by food antigens and migrate to the nearby mesenteric lymph node, where antigen-specific T cells are developed. These T cells then enter the systemic circulation and migrate to the peripheral lymph nodes and to distant tissues^33,43^. Alternatively, a small number of mucosal DCs migrate to distance sites by themselves, especially, to transport live gut bacteria^12^. To observe the holistic effects of mucosal immunity, DCs could be cultured in our co-culture system and moved to a separate culture of T cells or target tissues. Furthermore, many more additional types of cells could be co-cultured by juxtaposing respective cell inserts within a single vessel. These new techniques would help us better understand signaling within the mucosal community and the impact on the whole body.

## Materials and methods

### Cell lines

Caco-2 human colon epithelial cells were obtained from American Tissue Culture Collection (ATCC, CAT#: HTB-37, Manassas, VA). THP-1 human monocytic cells were obtained from ATCC (CAT#: TIB-202). HT29-MTX-E12 cells, mucous-secreting human intestinal cells, were obtained from Millipore Sigma (CAT#: 12040401, Burlington, MA). *Bacillus subtilis* (3A1^T^ (wild-type)) strain was obtained from Bacillus Genetic Stock Center (Ohio State University, Columbus, OH). All cell lines were authenticated by the suppliers and obtained with the appropriate Material Transfer Agreement. Mycoplasma testing of human cells gave negative results.

### Cell culture media

THP-1 cells were maintained at a cell density of 1 × 10^5^/mL to 1 × 10^6^/mL in RPMI 1640 (ThermoFisher Scientific, CAT#: 11875119, Waltham, MA) supplemented with 10% FBS, 1% GlutaMax (ThermoFisher Scientific, CAT#: 35050061), 10 mM HEPES buffer (Thermo Fisher Scientific, CAT#: 15630080), 4.5 g/L Glucose (Thermo Fisher Scientific, CAT#: A2494001), 1 mM Sodium Pyruvate (Thermo Fisher Scientific, CAT#: 11360070), and 1% penicillin/streptomycin (ThermoFisher Scientific, CAT#: 15140122) as described^44,45^. Caco-2 and HT29-MTX-E12 cells were cultured in DMEM with high glucose (4.5 g/L, ThermoFisher Scientific, CAT#: 11965092) and 1% GlutaMax (ThermoFisher Scientific, CAT#: 35050061) supplemented with 10% heat-inactivated FBS and 1% penicillin/streptomycin as described^46^. Caco-2 cell medium was also supplemented with 1% MEM Non-Essential Amino Acids Solution (Thermo Fisher Scientific, CAT#: 11140050). HT29-MTX medium was supplemented with 10 mM HEPES (Thermo Fisher Scientific, CAT#: 15630080). *Bacillus subtilis wt* (3A1^T^) strains were obtained from Bacillus Genetic Stock Center (https://bgsc.org/). They were cultured in LB medium at 37 °C with constant shaking at 200 rpm as previously described^47,48^.

### Reagents

The following reagents were used. l-Sepiapterin (SEP, Career Henan Chemical Co., CAS#: 17094-01-8, National University Science Park, High-Tech Zone, Zhengzhou City, China); PureCol Bovine Collagen (Advanced BioMatrix, CAT# 5005-100ML, Carlsbad, CA, USA, 1:30 dilution in ddH_2_O); EMD Millipore™ Poly-d-Lysine Solution (EMD Millipore, CAT# A003E, Burlington, MA, 1 mg/mL in ddH_2_0); LB Broth, Miller (Fisher Scientific, CAT#BP1426-2); PBS (10× PBS, Fisher Scientific, CAT# SH3037803); Albumin Bovine (Sigma, CAT#A-2153, as 1% in PBS); Normal Goat Serum (Fisher Scientific, CAT#16210072, as 10% in PBS); DAPI (Sigma, CAT#D9542-1MG); Triton X-100 (Sigma CAT#T8787-100ML, used as 0.1% in PBS); VECTASHIELD Mounting medium (Vector Laboratories, CAT#H-1000, Newark, CA, USA); phorbol 12-myristate 13-acetate (PMA, Invivogen, CAT CODE tlrl-pma, San Diego, CA, USA, 100 ng/mL); lipopolysaccharide (LPS, Sigma-Aldrich, CAT# L4391-1MG, 5 ng/mL), Interferon-γ (IFN-γ, PeproTech, CAT# 300-02, Rocky Hill, NJ, USA, 20 ng/mL), interleukin-4 (IL-4, PeproTech, CAT# 200-04, 20 ng/mL) and interleukin-13 (IL-13, PeproTech, CAT# 200-13, 20 ng/mL).

### Antibodies

The following antibodies were used. Anti-human MUC2 (996/1) (ThermoFisher, CAT# MA5-12345); Anti-human CD68 (Novus Biologicals, CAT# NB100-683-0.1 mg, Centennial, CO, USA); Anti-human CK20 (Life Technologies, CAT# PA5-82875); Anti-human CD80 (Cell Signaling Technologies, E3Q9V, CAT# 15416, Danvers, MA, USA); Anti-human CD 163 (Abcam, CAT# 156769, Cambridge, UK); Alexa Fluor 488 Goat anti-Rabbit IgG (ThermoFisher, CAT#A11008); Alexa Fluor 594 Goat anti-Mouse IgG (ThermoFisher, CAT#A11005).

### In vitro macrophage polarization

THP-1 cells were subjected to differentiation followed by M1 vs. M2 polarization as described^49^. Briefly, cells were seeded in 24-well plates at a density of 250,000 cells/mL. To differentiate monocytic THP-1 cells to macrophages, cells were treated with 100 ng/mL PMA for 24 h. To obtain M1-polarized macrophages, 5 ng/mL LPS and 20 ng/mL IFN-γ were added to PMA-treated cells, and cells were maintained for up to 66 h. To obtain M2-polarized macrophages, 20 ng/mL IL-4 and 20 ng/mL IL-13 were added to PMA-treated cells, and cells were maintained for up to 66 h. M1- or M2-polarization was confirmed by the immunofluorescence staining against CD80 vs. CD163 as we routinely performed^19^. For macrophage reprogramming experiment, 100 μM SEP was added to M2-polarized macrophages, and cells were maintained for 3 days. Medium was unchanged throughout the entire differentiation-polarization-reprogramming experiment.

### Construction of 3D-Flipwell for gut mucosal co-culture systems

A custom insert was constructed as follows. Two BRAND Inserts for 12-well plates PET-membrane 0.4 µm (BrandTech Scientific Inc., CAT# 782730, Essex, CT, USA) were used to construct a “3D-Flipwell” (Figs. 2, 3). All work was performed under sterile conditions inside a cell culture cabinet (Class II A/B3 Biological Safety Cabinet 1300 Series A2, ThermoScientific). The membrane in one of the inserts was removed with a #10 sterile scalpel blade (Kent Scientific Inc., CAT#INS500348, Torrington, CT, USA). Loctite Clear Silicone Waterproof Sealant (aquarium-safe glue, Henkel Corp., CAT#: ASTM D4236, Düsseldorf, Germany) was applied around the bottom edge of one of the inserts. The inserts were then joined bottom-to-bottom with a slight pressure, such that the L-shaped rim hooks were positioned at a 180 degrees from each other, and left to cure overnight. UV irradiation treatment of 1 h for each side of the Flipwell was adequate to ensure no contamination. To ensure there was no leak in the system, each side of inserts was filled with sterile PBS and left upright for one hour to check any leak. To ensure cell adhesion, the membrane was coated, one side at a time, with a thin layer of 3:2 PureCol (1:30 dilution) to Poly-d-Lysine (50 µg/mL) in ddH_2_O and left at RT for 2 h, washed with sterile PBS twice, flipped over, and the opposite side of the membrane was coated and washed. We used a 100 × 25 mm deep polystyrene stackable petri dish bottom (USA Scientific, CAT# 8609-0625, Ocala, FL, USA) with a wider TC plate lid (Corning 100 mm × 20 mm Style Dish, Corning, CAT #430167, Corning, NY, USA) for this work. The Flipwells were hung from the rim of the petri dish and covered with the Corning TC plate lid. Once the membrane coating dried, the 3D-Flipwells were ready to be used for the downstream co-culture assays (Figs. 2, 3). To confirm the lack of negative impact of chemicals used to assemble the Flipwells, 2–3 wells were seeded with Caco2 cells, and intact cell growth was observed.

### 3D co-culture using the Flipwells

The topside of the insert membrane was seeded with the colon epithelial Caco2 cells and HT-29-MTX enterocytes at a 9:1 ratio at the density of 7.5 × 10^4^/cm^2^ per protocol previously described^46^ and cultured for 7–10 days to ensure polarization of gut epithelium and establishment of overlying mucus (Fig. 2). The insert stack was then flipped over, and THP-1 cells were seeded at 0.5 × 10^6^ cells on the underside of the membrane with PMA (100 ng/µL) for 24 h to ensure monocyte adhesion/macrophage differentiation (Fig. 2). After THP-1 cells were attached to the membrane, the insert stack was flipped back to have the colon epithelial cells on the topside and macrophage on the underside of the membrane (Fig. 2). Co-cultures were maintained for 2–3 weeks with medium change every other day until mucus was formed over colon epithelial cells. The Flipwells were further subjected to bacterial co-culture, drug treatment and confocal and electron microscopy (see below).

### Addition of bacteria to the Flipwell

BrandTech Brand Insert for 24 well plates PET-membrane, 0.4 µm (BrandTech, CAT #782710) was used for bacterial culture added to the Flipwell co-culture. To ensure the inserts will fit inside the 12-well-sized Flipwells, the 3 little feet at the bottom of the 24-well insert were broken off using sterile tweezers under sterile conditions, and the inserts were UV-irradiated for 1 h to ensure no contamination. 500 µL media was removed from the Flipwell to accommodate the bacterial insert. 500 µL of *B. subtilis* bacteria in cultured in LB media (10^14^ CFU) plus SEP 100 µM was added to the 24-well insert into the Flipwell and hung from the Flipwell rim and incubated for 3 h (Figs. 2, 3).

### SEP treatments to induce nitric oxide production

To assess the effects of NO production on the gut mucosal microenvironment using our novel co-culture system, SEP was added at the final concentration of 100 µM to the bacterial insert, the gut epithelial side of the insert stack and co-culture medium (Figs. 2, 3). After removing bacterial insert, co-cultures were further treated with SEP for 3 days with medium change everyday. Co-cultures were processed for SEP and confocal imaging.

### Immunofluorescence staining and confocal microscopy

The Flipwells were washed in 15 mL PBS inside a 50 mL conical tube. The cells were fixed with 15 mL 4% PFA in PBS for 10 min inside a 50 mL conical tube, washed with 15 mL PBS and processed for immunostaining or alternatively left in 15 mL of 0.4% PFA in PBS overnight for downstream applications. To stain both sides of the Flipwell membrane, the inserts were separated by hand with a twist action, and silicone gasket ring removed with tweezers. The gut epithelial side of the membrane was permeabilized with 0.1% Triton-X 10 min and washed with PBS 2× times. Both sides of the membrane were blocked in 10% Normal Goat Serum in PBS 30 min RT, and primary antibodies applied at a 1:100 ratio in 1% BSA in PBS overnight at 4 °C. It is important to note, that one side of the membrane rested on a large drop placed on top of Saran plastic food wrap of an antibody-1% BSA in PBS mixture, while the other side still had the insert walls to retain the antibody-1% BSA in PBS mix. The membranes were washed in PBS 3 × 10 min, and secondary antibodies were applied at 1:1000 at RT 1 h. The membranes were washed again 3× times in PBS 10 min, and DAPI applied at 1:100 10 min RT and kept in dark. The membranes were cut out with a sterile scalpel blade and placed on a round cover slip (Fisherbrand Microscope Cover Glass, CAT#12-546-2 25CIR-2) with a few drops of the mounting medium on each side and covered with the second glass coverslip to make a sandwich-like assembly. The assembly was affixed between two metal rings of the Attofluor cell chamber (ThermoScientific/Life Technologies, CAT#A7816) for confocal imaging with Leica Microsystems TCS SP5 multi-photon laser scanning confocal microscope using Suite Advanced Fluorescence (LAS AF) software*.*

#### Image analysis

Quantification of fluorescence signal in micrographs was performed with ImageJ software (NIH) referring to the owner’s manual (http://imagej.net/docs/guide/146.html). To determine the percent positive cells, the threshold levels (minimum and maximum cut offs) of each antigen signal were determined using a control image, and the same range was applied to the remaining control and treatment images. Each image was divided into four quadrants, and the number of positive cells in each quadrant was counted. Percent positivity was determined with respect to the number of DAPI-stained nuclei. To determine the average intensity of each antigen, regions of interest (ROI) encompassing 5–10 cell cluster were chosen, and the mean intensity for each ROI was measured. For each image, 5–10 ROIs were chosen, leading to 20–30 measurements for each sample set. The statistical significance of the data was evaluated using Graphpad Prism Version 9 software (see “Statistics” section).

### Scanning electron microscopy (SEM)

The Flipwells (seeded with Caco2:HT29-MTX cells on one side and THP-1 cells on the other side) were treated with SEP and bacterial metabolites (inside the insert). The membranes were fixed in 4% paraformaldehyde, washed twice with 10× phosphate buffer saline and then DI water. Chemical drying was performed using increasing concentrations of ethanol in water (25%, 50%, 70%, and 100%), followed by washing each sample with a 50% Hexamethyldisilazane (HMDS): Ethanol solution, and then with 100% HMDS. Each membrane was removed from the wells with forceps, placed onto a piece of weighing paper, and cut in half with a scalpel. One side of the membrane was bound with carbon tape to the aluminum SEM sample stub with the top facing up. The remaining side was flipped over and bound to the stub with the bottom facing up. All samples were sputter coated with gold for 15 s using a Denton Vacuum Desk II Cold Sputter unit. The coated samples were imaged in the JEOL 7500F Scanning Electron Microscope at an acceleration voltage of 2 kV using the LEI detector.

#### Statistics

All the experiments were performed in replicates (n ≥ 3), and measurements of samples were performed in 20–30 readings, ensuring the adequate statistical power as done previously^50^. Unless otherwise indicated, statistical significance of the mean difference was tested by two-tailed t-tests (parametric) using GraphPad Prism Version 9 software. p-values of 0.05 or less were considered significant.

## Supplementary Information

Supplementary Information.

## Data Availability

All data generated or analyzed during this study are included in this published article [and its Supplementary Information file].
