# Supplementary material for: Novel 3D Flipwell system that models gut mucosal microenvironment for studying interactions between gut microbiota, epithelia and immunity
Source: Sci Rep. 2023 Jan 17;13:870. doi: 10.1038/s41598-023-28233-8 (PMC9845379; doi:10.1038/s41598-023-28233-8)

Supplementary Information

1. Original SEM Images

A. THP-1 (mono-cultured M0, M1 & M2)

i) Control (Mock)-treated

a. M0 (Naïve)

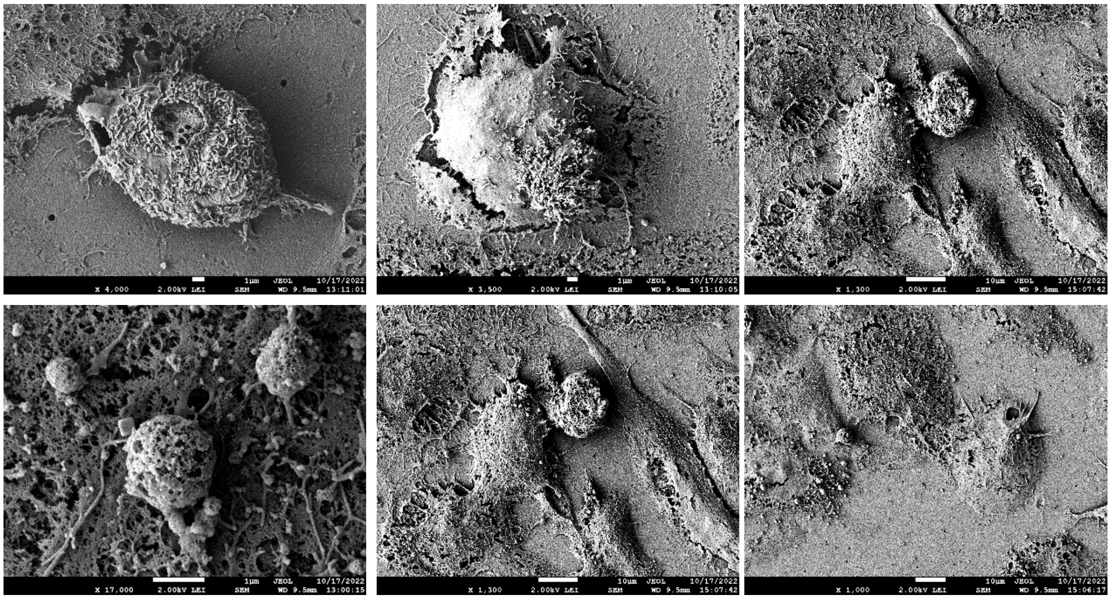

b. M1

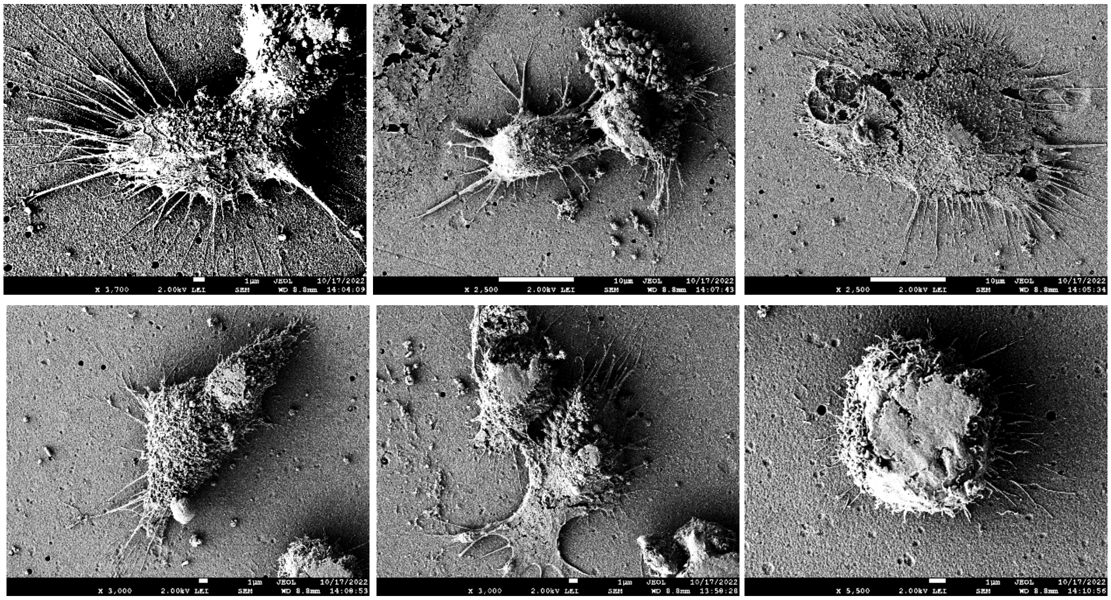

c. M2

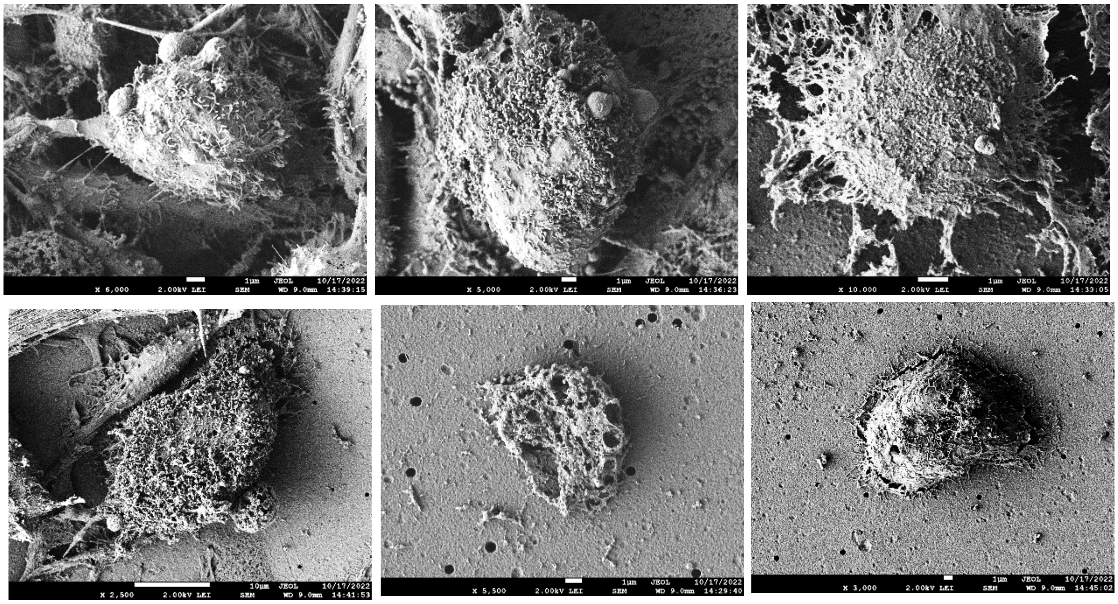

ii) SEP (100 μM)-treated

d. M0 (Naïve) + SEP

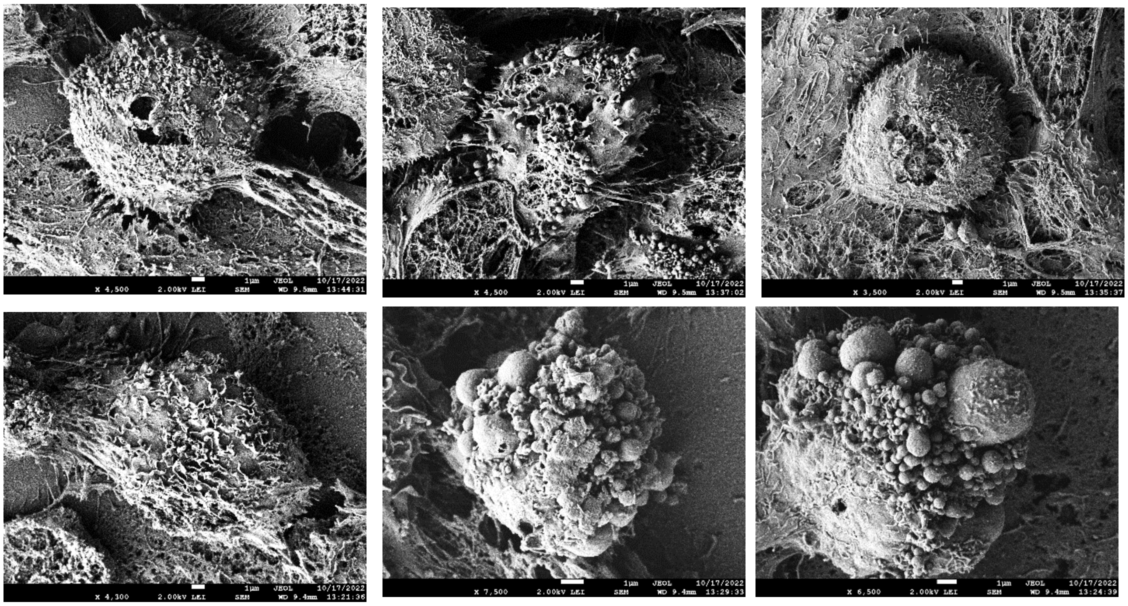

e. M1 + SEP

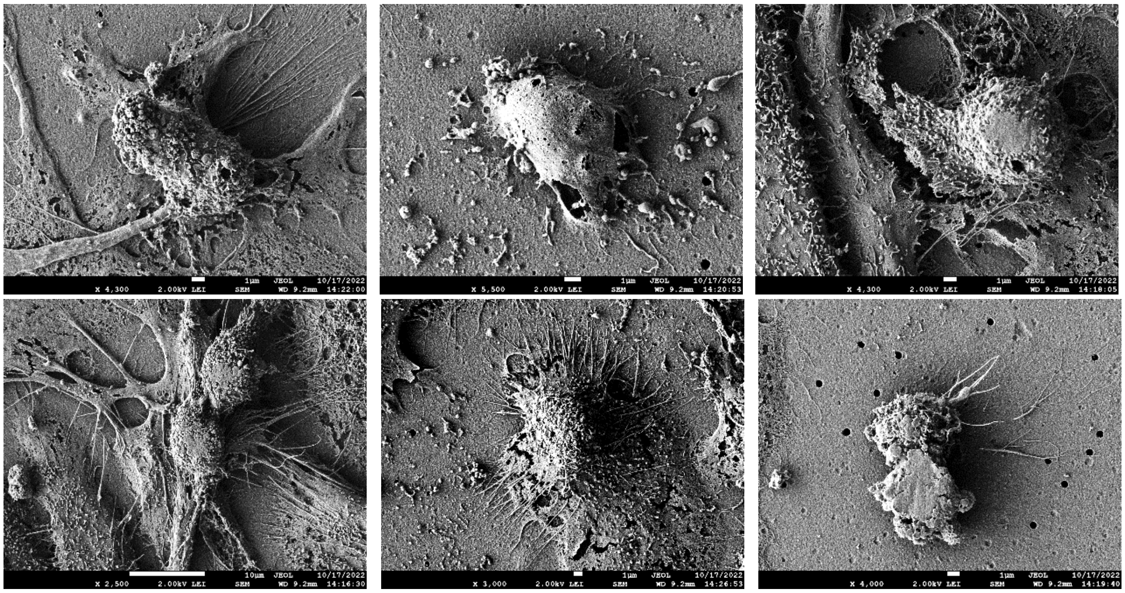

f. M2 + SEP

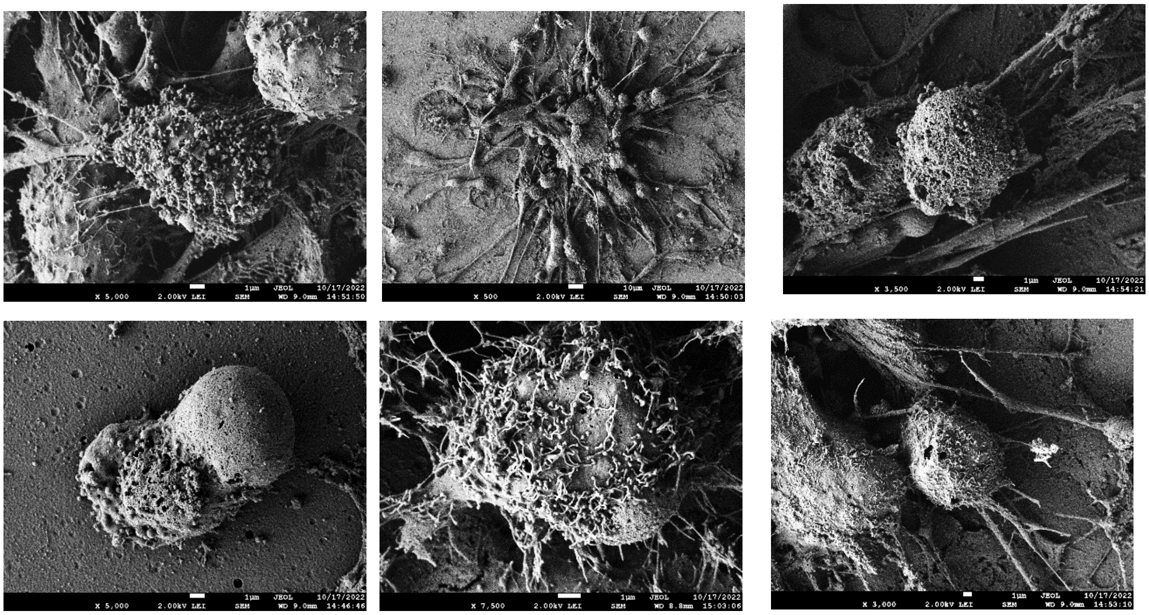

B. Bacillus subtills (mono-cultured)

i) Control (Mock)-treated

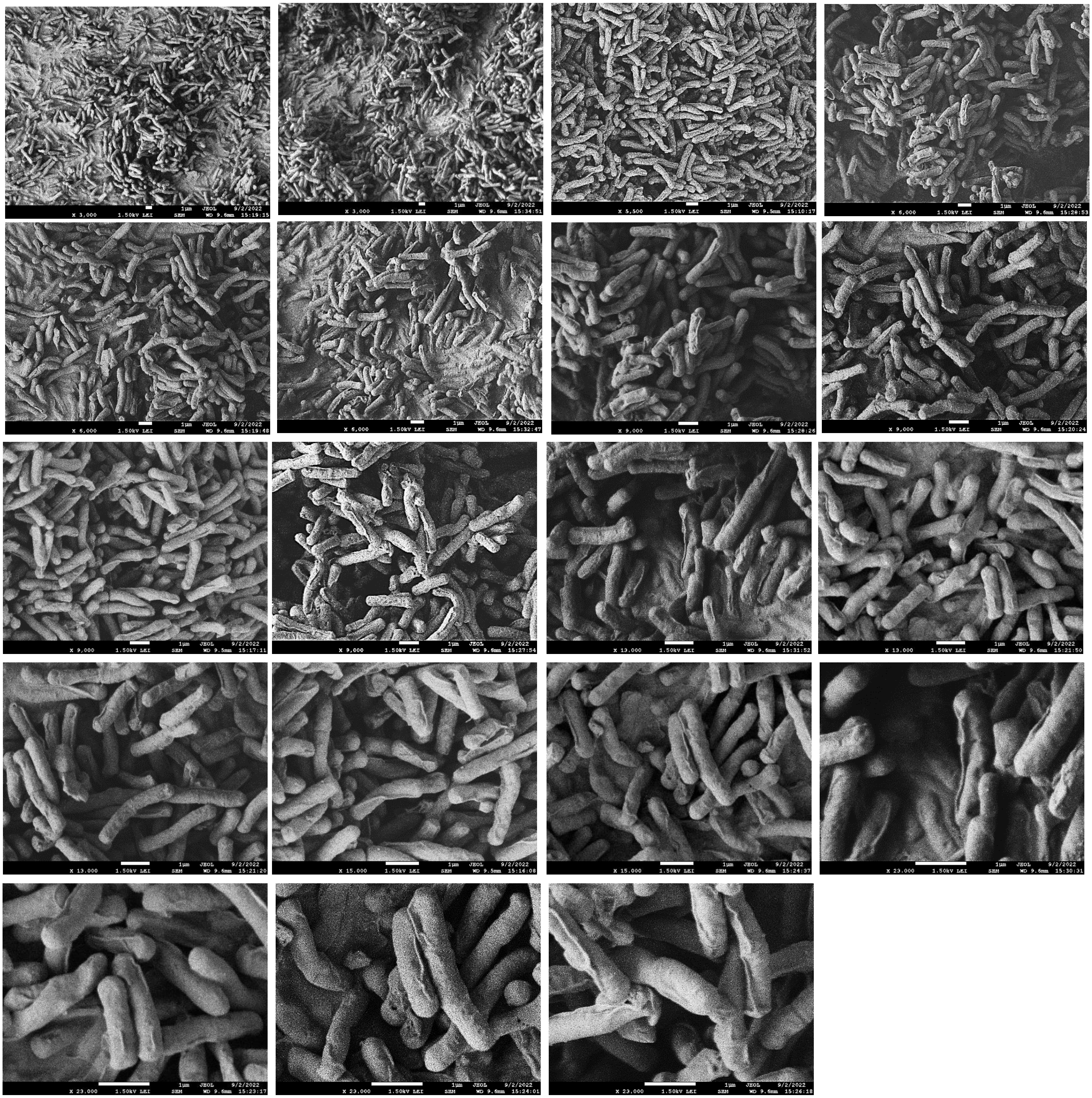

ii) SEP (100 μM)-treated

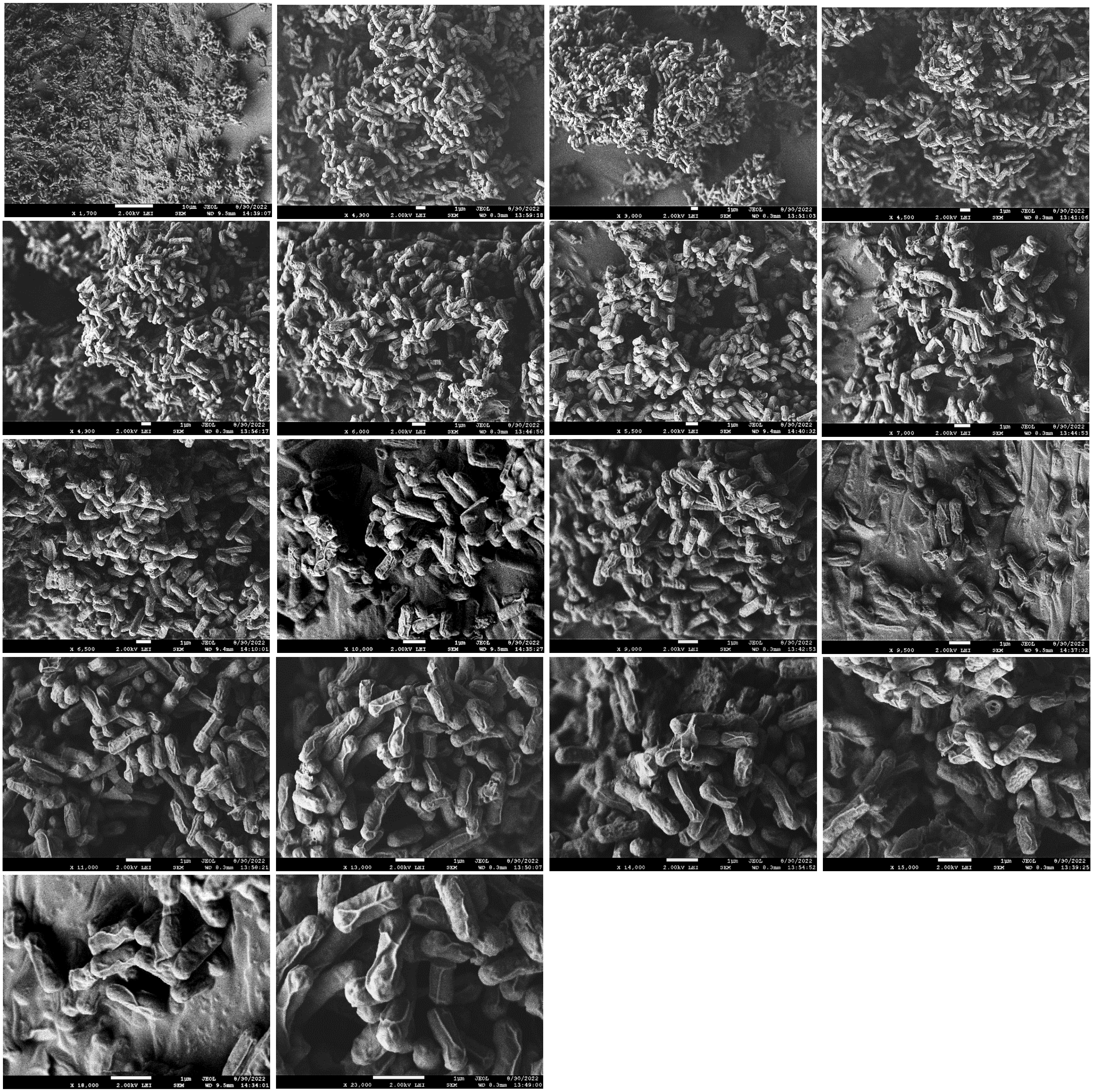

B. Caco-2/HT-29 (co-cultured)

i) Control (Mock)-treated

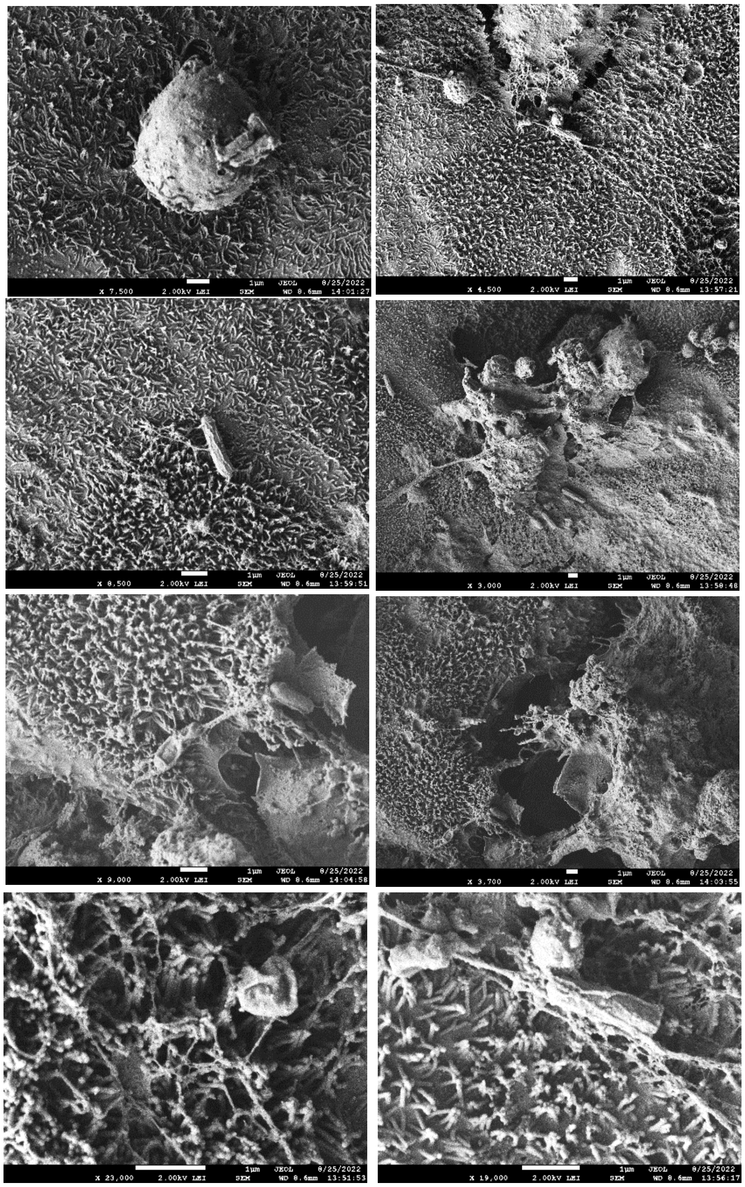

ii) SEP (100 μM)-treated

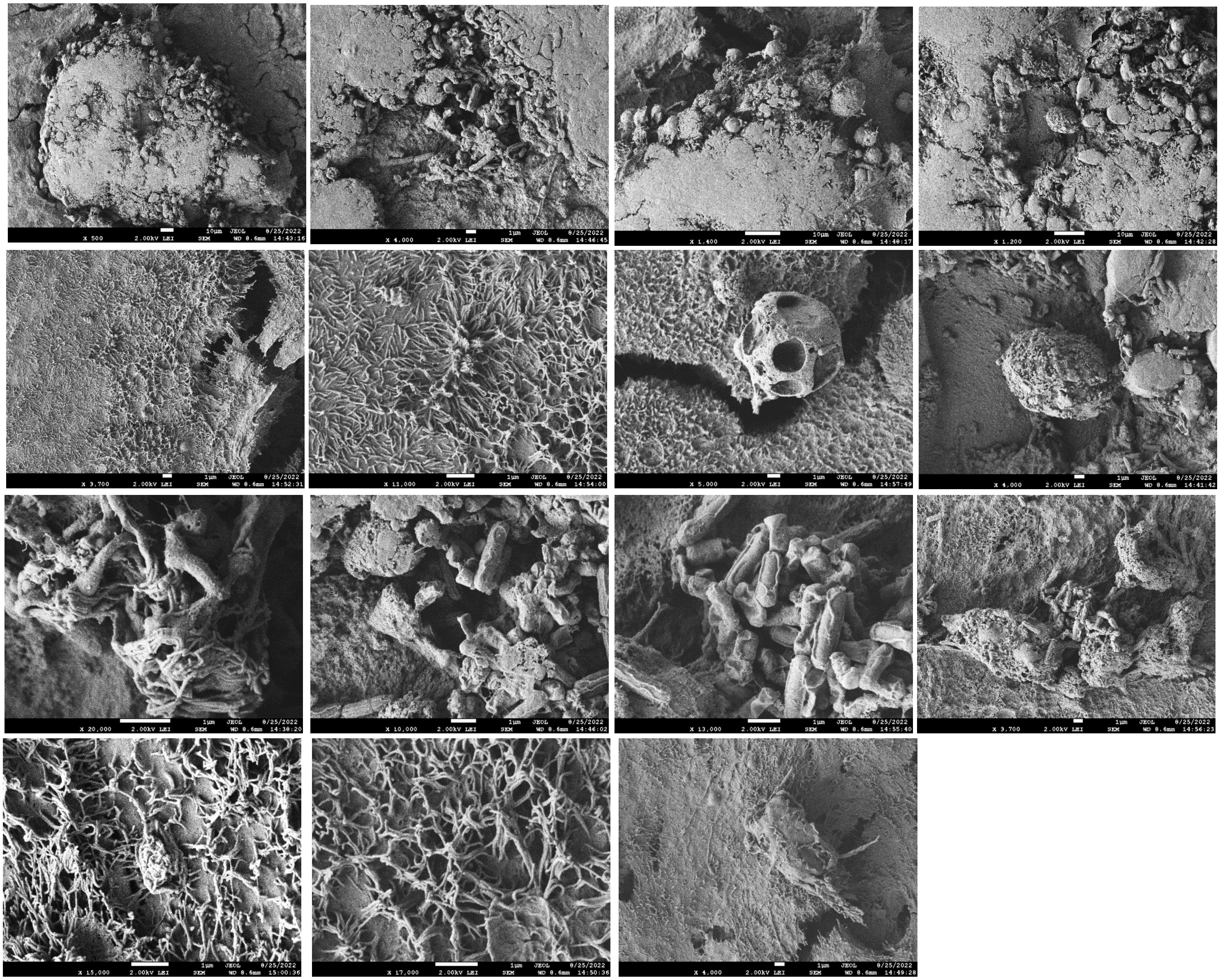

C. THP-1 M0 (co-cultured)

i) Control (Mock)

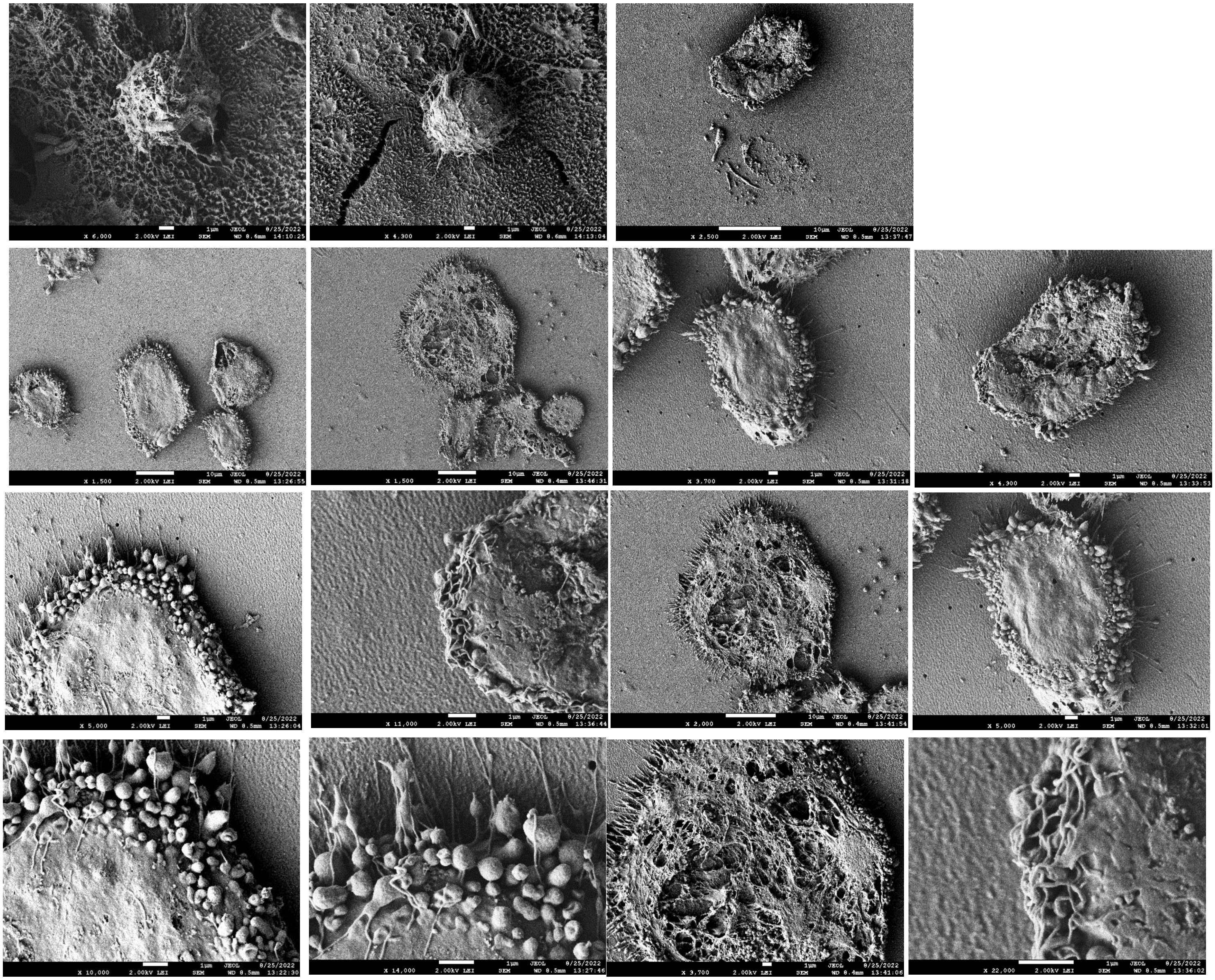

ii) SEP (100 μM)

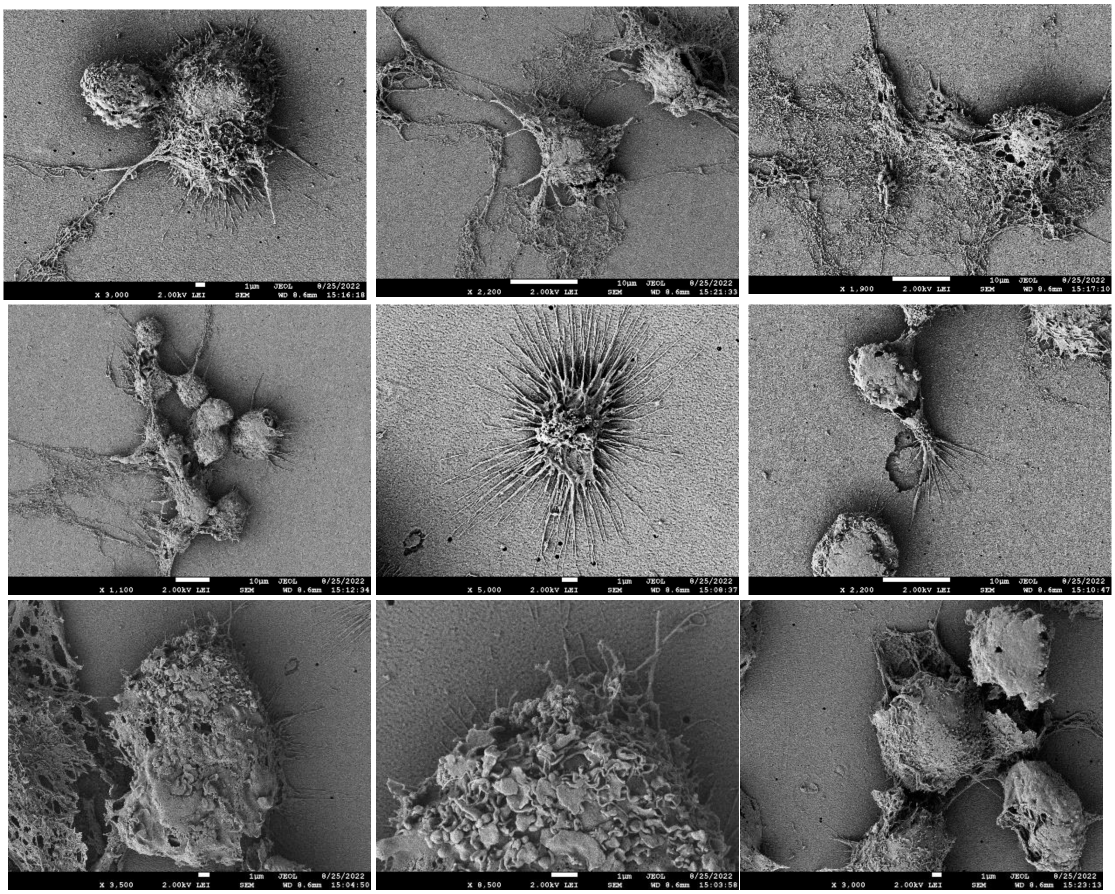
1μm JEOL 8/25/2022
X 3,000 2.00kV LEI SEM WD 8.6mm 15:16:18
10μm JEOL 8/25/2022
X 2,200 2.00kV LEI SEM WD 8.6mm 15:21:33
10μm JEOL 8/25/2022
X 1,900 2.00kV LEI SEM WD 8.6mm 15:17:10
10μm JEOL 8/25/2022
X 1,100 2.00kV LEI SEM WD 8.6mm 15:12:34
1μm JEOL 8/25/2022
X 5,000 2.00kV LEI SEM WD 8.6mm 15:08:37
10μm JEOL 8/25/2022
X 2,200 2.00kV LEI SEM WD 8.6mm 15:10:47
1μm JEOL 8/25/2022
X 3,500 2.00kV LEI SEM WD 8.6mm 15:04:50
1μm JEOL 8/25/2022
X 8,500 2.00kV LEI SEM WD 8.6mm 15:03:58
1μm JEOL 8/25/2022
X 3,000 2.00kV LEI SEM WD 8.6mm 15:23:13

2. Original Confocal Images

A. Caco-2/HT-29
i) Control (Mock)

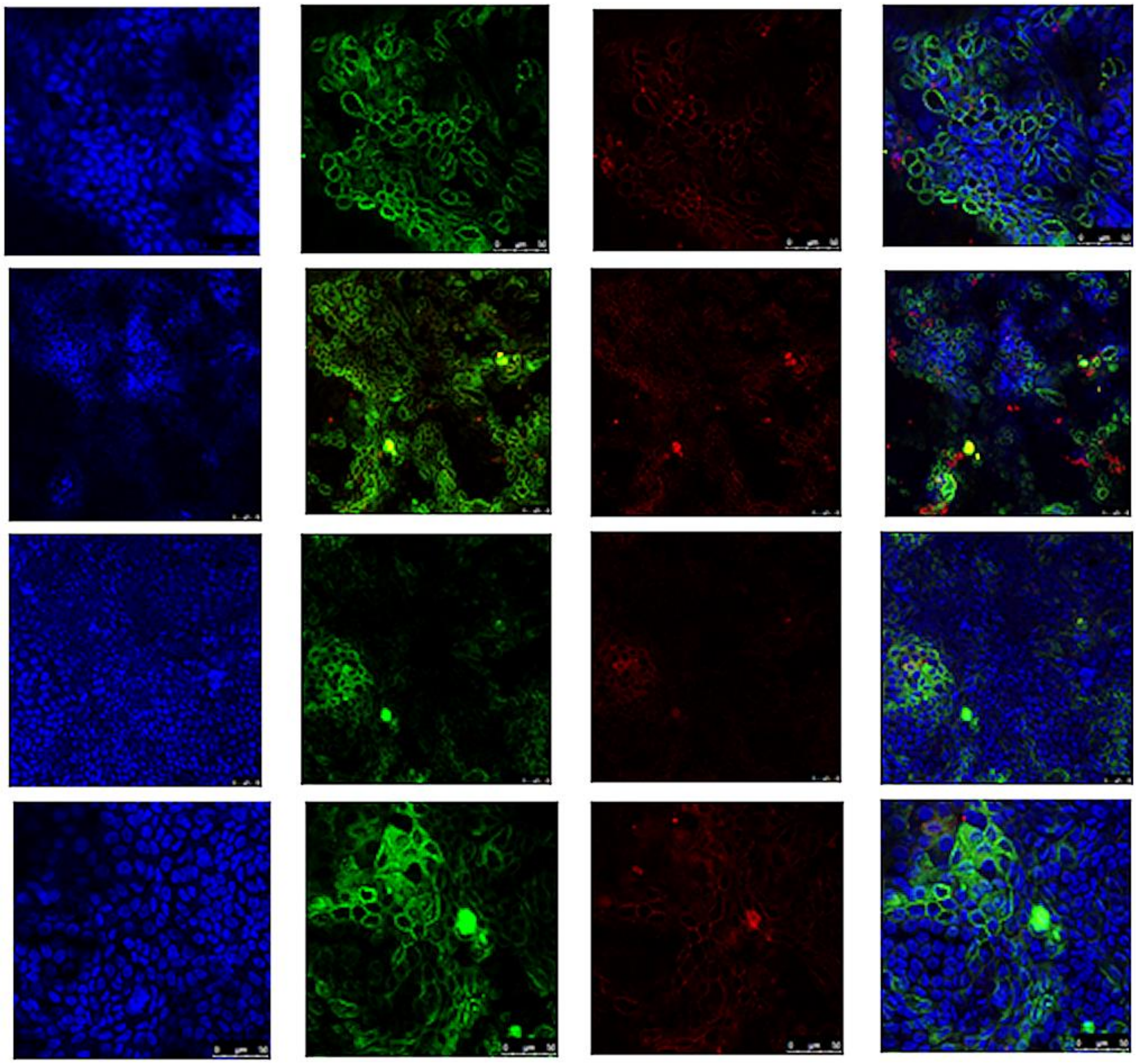

ii) SEP (100 μM)

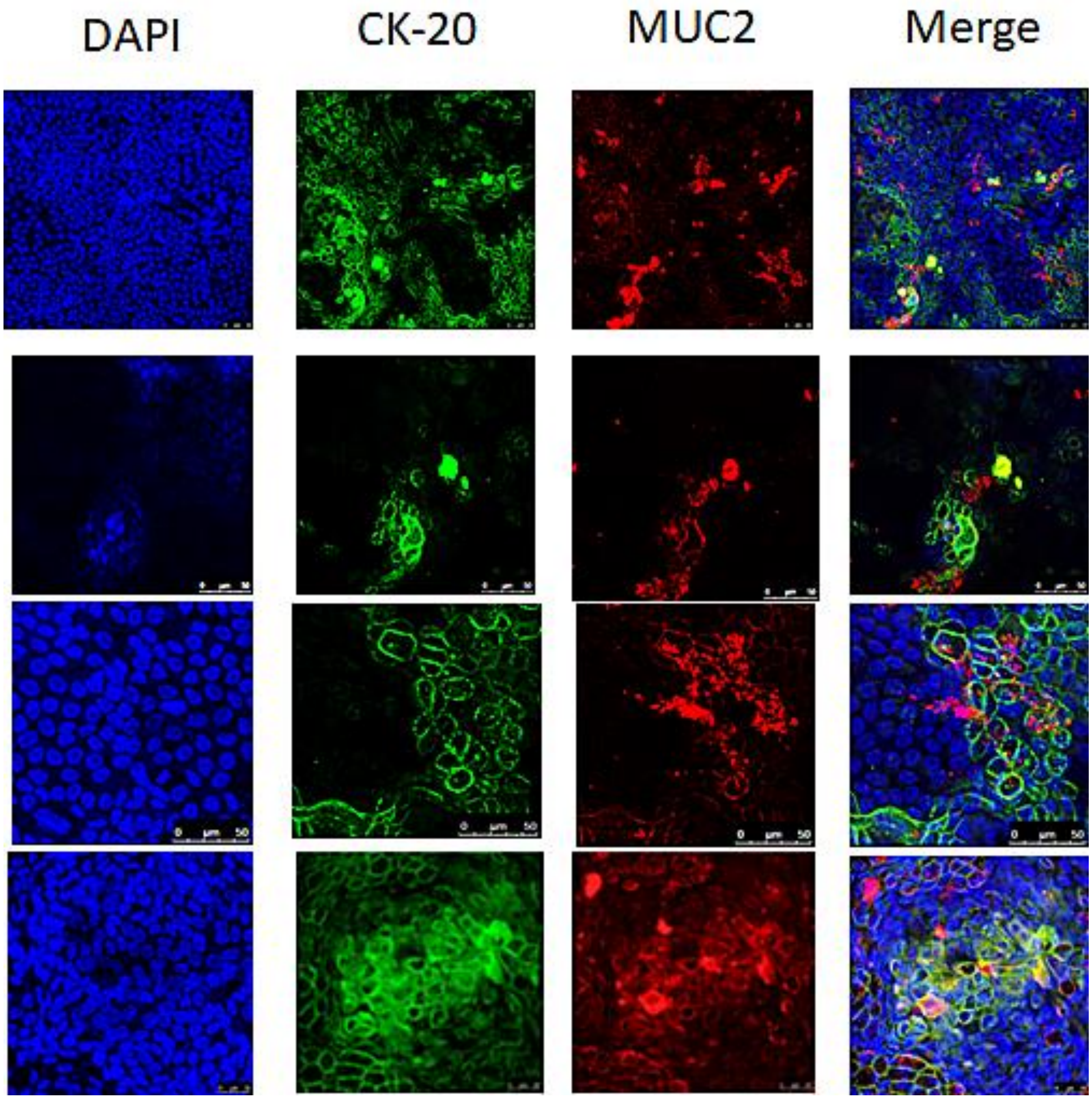

B. THP-1

i) Control (Mock); CD80/CD68

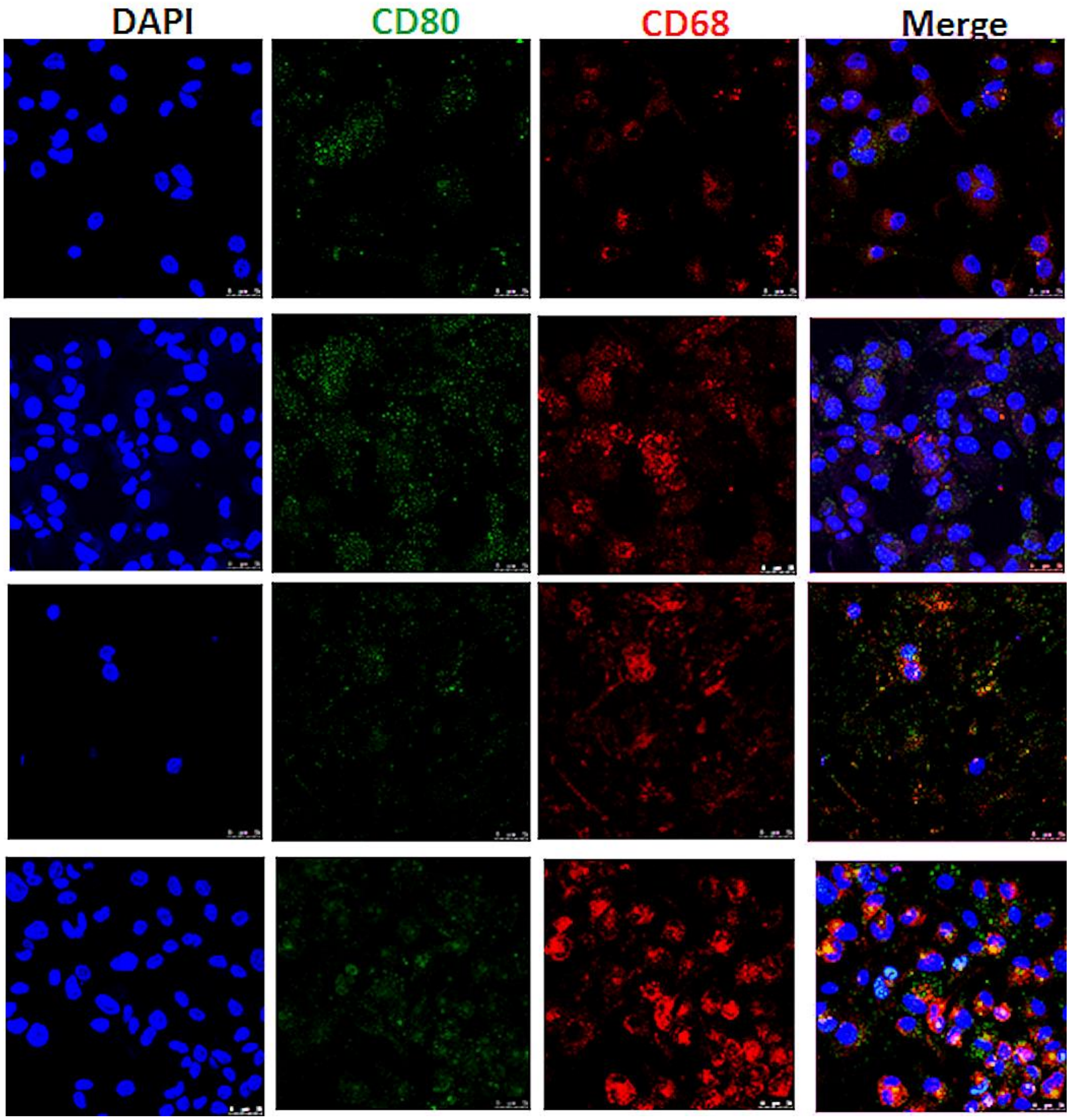

ii) SEP (100 μM)); CD80/CD68

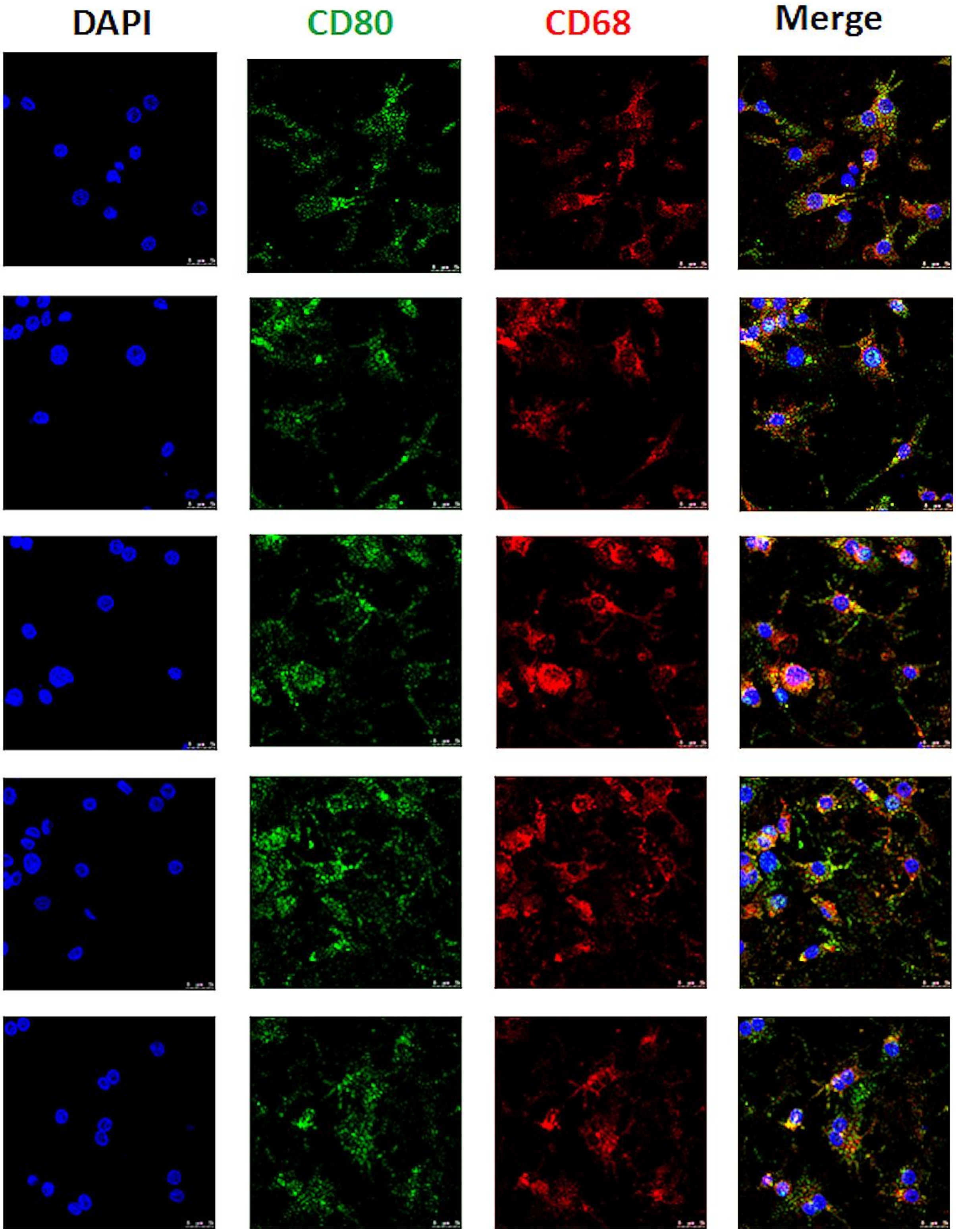

iii) Control (Mock); CD80/CD163

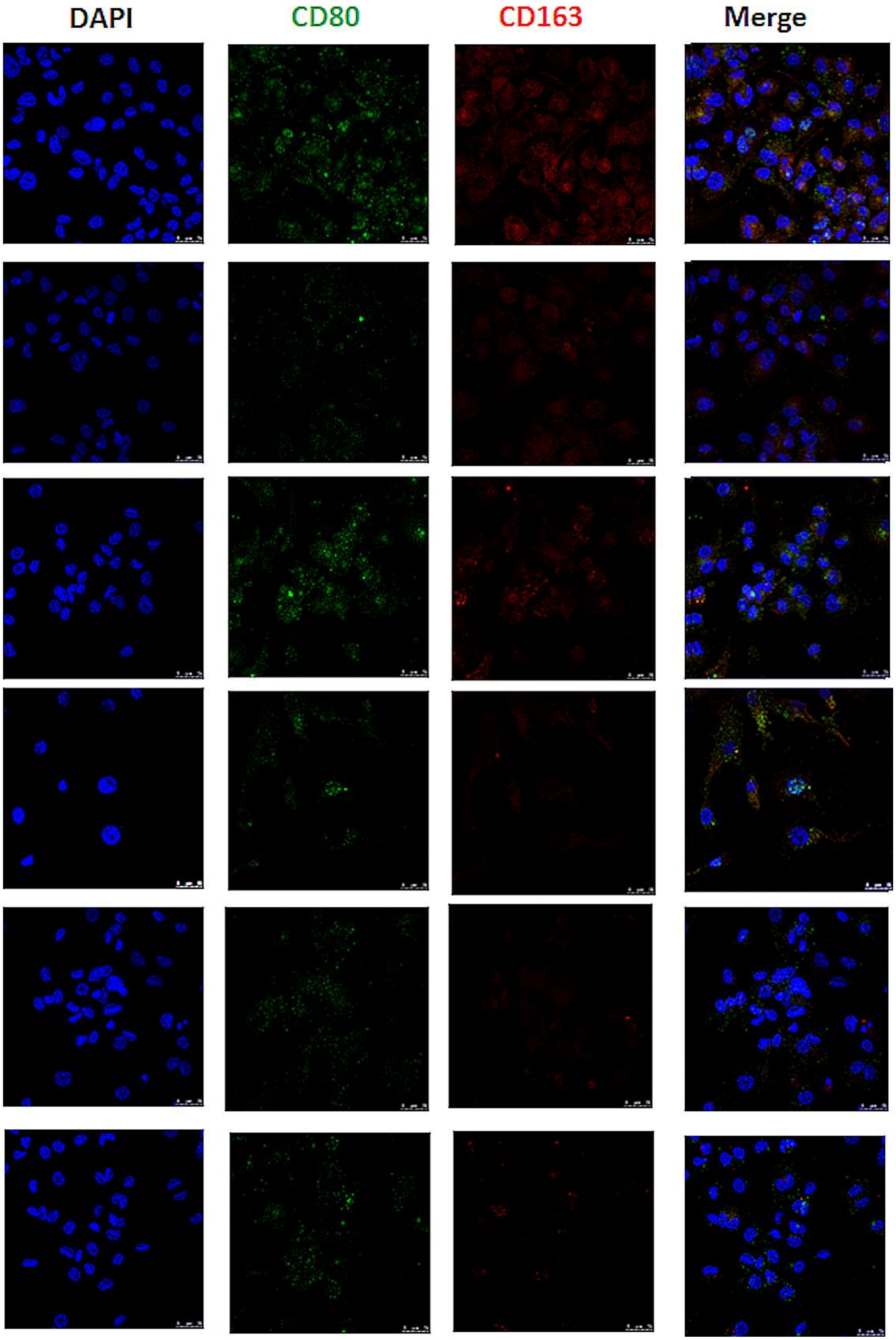

iv) SEP (100 μM) CD80/CD163

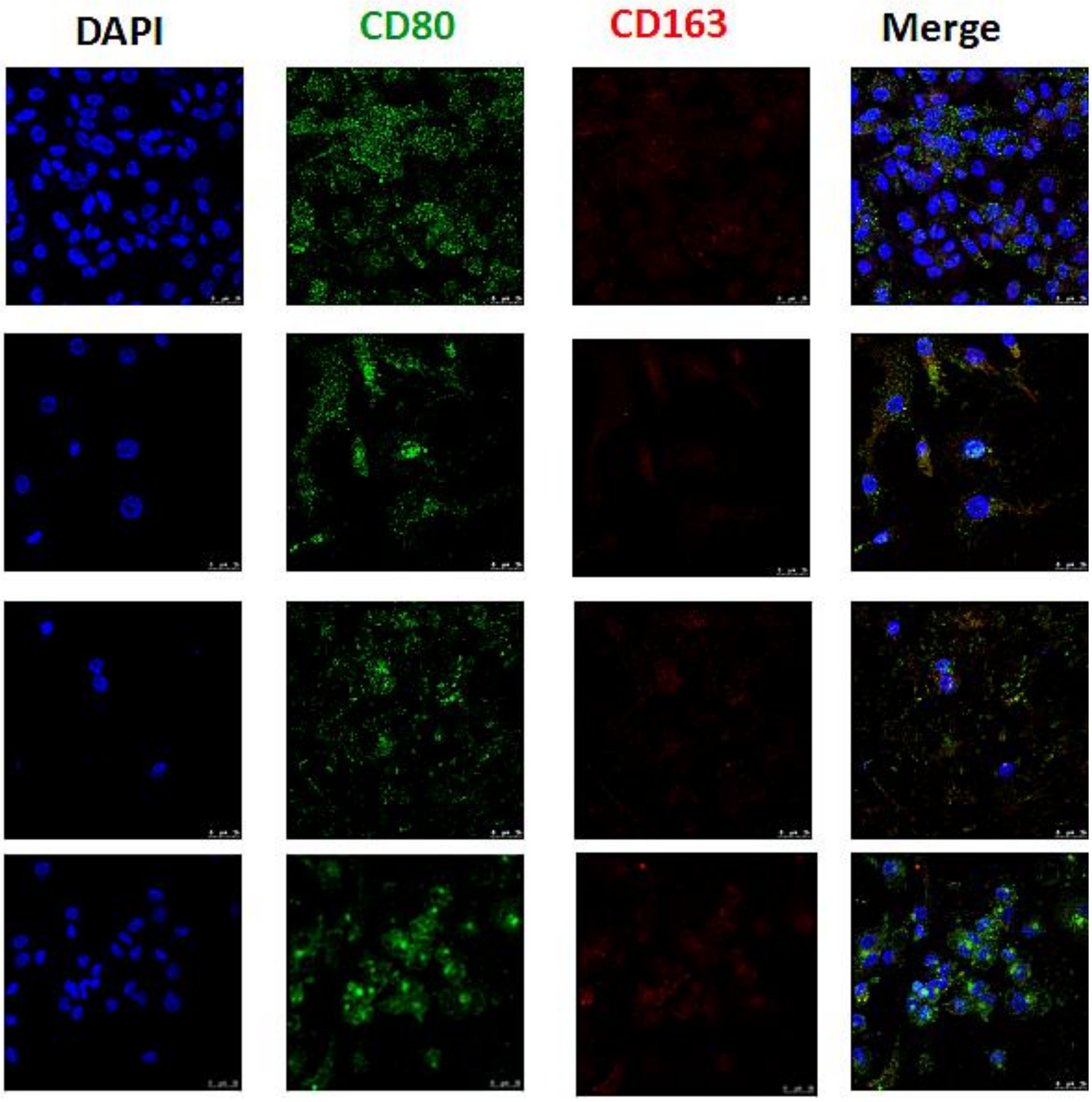

Supplement: Supplementary file 1 — Supplementary Information. [file 41598_2023_28233_MOESM1_ESM.pdf]
